# *Nanoscale zerovalent* copper (nZVC) catalyzed environmental remediation of organic and inorganic contaminants: A review

**DOI:** 10.1016/j.heliyon.2022.e10140

**Published:** 2022-08-08

**Authors:** Sandeep Kumar, Parminder Kaur, Ravinderdeep Singh Brar, J Nagendra Babu

**Affiliations:** a Department of Chemistry, Akal University, Talwandi Sabo, Bathinda, 151302, Punjab, India; b Department of Chemistry, School of Basic and Applied Science, Central University of Punjab, Bathinda, 151001, Punjab, India

**Keywords:** *Nano zerovalent* copper, Environment remediation, Redox process, Dyes, Drugs, Pharmaceuticals, Heavy metals

## Abstract

Over the past decade, the nano zerovalent copper has emerged as an effective nano-catalyst for the environment remediation processes due to its ease of synthesis, low cost, controllable particle size and high reactivity despite its release during the remediation process and related concentration dependent toxicities. However, the improvised techniques involving the use of supports or immobilizer for the synthesis of Cu^0^ has significantly increased its stability and motivated the researchers to explore the applicability of Cu^0^ for the environment remediation processes, which is evident from access to numerous reports on nano zerovalent copper mediated remediation of contaminants. Initially, this review allows the understanding of the various resources used to synthesize zerovalent copper nanomaterial and the structure of Cu^0^ nanoparticles, followed by focus on the reaction mechanism and the species involved in the contaminant remediation process. The studies comprehensively presented the application of nano zerovalent copper for remediation of organic/inorganic contaminants in combination with various oxidizing and reducing agents under oxic and anoxic conditions. Further, it was evaluated that the immobilizers or support combined with various irradiation sources originates a synergistic effect and have a significant effect on the stability and the redox properties of nZVC in the remediation process. Therefore, the review proposed that the future scope of research should include rigorous focus on deriving an exact mechanism for synergistic effect for the removal of contaminants by supported nZVC.

## 1 Introduction

Growing demands of industrial goods with the population boom has resulted in an increased discharge of industrial effluents into water streams and soil. In addition, many natural and anthropogenic activities also result in contamination of terrestrial and aquatic environments. These activities release effluents of organic/inorganic origin, which pose serious threats to flora and fauna and have adverse physiological effects on humans and ecosystem (Mahmoud and Gan, 2018). So, there is an urgent need to develop sustainable methods and technologies to treat the municipal waste and industrial effluents enormously loaded with organic and inorganic contaminants. In the past few decades, many conventional and economically non-viable approaches including physical, chemical and biological process have been employed for the remediation of the environment, but the real solution to this problem has been provided by nanotechnology in the form of tailored nanoparticles (Akhtar et al., 2020). The nanoscale zerovalent nanoparticles such as iron (Fe^0^), cobalt (Co^0^), nickel (Ni^0^), copper (Cu^0^) and zinc (Zn^0^) etc. owing to their distinct physico-chemical properties and the significant role they played in the removal of various contaminants, have attracted the attention of a large scientific community (Li et al., 2016). The high contaminant removal efficiencies of these nanoscale materials are associated with their high surface area to weight ratios, which renders them high reactivities towards various contaminants in comparison to their microscale metal counterparts (E Guerra et al., 2018). Among these easily available metallic particles, copper has been significantly evaluated in literature for its environment remediation processes due to high abundance, low cost, controllable particle size, high reactivity and tunable redox properties.

Copper (^63^Cu_29_) is an element derived from its ores like chalcopyrite (CuFeS_2_, 34.5% Cu), Cuprite (Cu_2_O, 88.8%), chalcocite (Cu_2_S, 79.8%), covellite (CuS, 66.5%), malachite (CuCO_3_.Cu(OH)_2_, 57.7%) etc. using various electrolytic refining processes (Daehn and Allanore, 2020; Moskalyk and Alfantazi, 2003). Since its discovery around 3500 BC, it found many application ranging from manufacturing of weapons and coins, as alloys with metals like tin (bronze), zinc (brass), Ag (sterling silver), Ni (cupronickel, constantan) for the making industrial goods etc (Davis, 2001; Powell and Webster, 2019). Due to its high thermal conductivity (401 W m^−1^ K^−1^ at 0 °C) and electro-conductivity (5.96 × 10^7^ S m^−1^ at 20 °C), it is also used in the manufacturing of thermocouples and electrical devices (Berman and Macdonald, 1952; Dadras and Morris, 1997; Lu et al., 2004; Maki et al., 2013; Minneci et al., 2021; Pickard et al., 2021). Further, with the emergence of field of nanochemistry, and due to its distinct variable oxidation states (Cu^0^, Cu^I^, Cu^II^, Cu^III^), copper based nanomaterial found many application in diverse areas such as catalysis viz. electrocatalysis, photocatalysis and organic transformation (Chen et al., 2021; Dong et al., 2019; Gawande et al., 2016; Ojha et al., 2017; Sambiagio et al., 2014; Shi and Buchwald, 2015), plant biology (Lamichhane et al., 2018; Liu et al., 2018; Panagos et al., 2018), medicine (Ahmedova et al., 2018; Mallick and Sabui, 2021) and environment remediation (de Sousa et al., 2019a; Fang and Guo, 2018). However, the pyrophoric nature of Cu^0^ nanomaterial due to its high sensitivity towards O_2_ and H_2_O restrict their application in various fields like optics and electronics etc (Gawande et al., 2016).

Recent studies have confirmed that the advanced techniques and improved methodologies comprising the involvement of immobilizers/stabilizers have enhanced the stability of Cu^0^ nanoparticles having core-shell structure (Ali et al., 2018; Huang et al., 2017; Issaabadi et al., 2017; Wang et al., 2017). The redox behavior of Cu^0^ is very useful in determining the stability and its surface passivation. The Cu^0^ has inherent stability at negative potential. Further, the oxidation of Cu^0^ is observed over a wide range of pH; with formation of Cu^+^ in acidic and neutral solutions and Cu^2+^ at higher pH values. The acidic pH favors the dissolution of Cu^0^ and formation of thermodynamically favorable species as a product; while the alkaline medium produces a layer of oxide over metal, thus preventing its further corrosion (Beverskog et al., 1997). Thus, the catalytic ability of Cu^0^ is related to generation of electron and ionic species (Cu^+^ and Cu^2+^), along with oxides and hydroxides over metal surface while performing the reductive degradation of environment contaminants (Yamaguchi et al., 2018). When present in its lowest oxidation state (Cu^0^) in the system, it has a strong tendency to involve in redox processes through one-electron transfer (Cu^+^ + e^−^ → Cu^0^, E^o^ = +0.521 V) or two-electron transfer (Cu^2+^ + 2e^−^ → Cu^0^, E^o^ = +0.342 V) (Oliveira et al., 2018) and thus catalyze a number of homogeneous oxidative coupling reactions (Ojha et al., 2017; Saha and Das, 2018; Sambiagio et al., 2014) or heterogeneous Fenton-like degradation processes (Lu et al., 2004). However, the homogeneous reaction has an associated disadvantage of catalyst consumption and sludge formation (Matavos-Aramyan et al., 2017).

Over the period the researchers have developed heterogeneous Fenton-like reaction systems involving the use of nano zerovalent copper as Fenton catalyst with oxidizing agents like H_2_O_2_ (Ma et al., 2018). The catalytic efficiency of nano zerovalent copper was significantly enhanced by the use of porous materials such as zeolite (Danish et al., 2017), green rust (Fang et al., 2019), iron oxide (Kim and Ko, 2018), carbon nanotubes (Zheng et al., 2021), cellulose (Kamal et al., 2019), chitosan Alani et al. (2021) etc. as an immobilizer or support. The catalytic performance of Cu^0^ is enhanced by catalyst’s support via adsorption of contaminants near the active sites and further assistance in generation of radical species and initiation of other pollutant decomposition pathways. In Fenton-like reactions, the rate of generation of free radical species is accelerated by the use of combination of Cu^0^ catalyst with ultrasound energy (Wang et al., 2019b), microwave radiations (Lee et al., 2010), ultraviolet radiation (Dinesh et al., 2020) along with oxidizing agents such as H_2_O_2_ (Liu et al., 2021b) and persulfate (Zhang et al., 2020b), or a combination of these.

Although there are several reports on the preparation of nano zerovalent copper and its application for the environment remediation process, however, no review paper has summarizes the effect of various dependent parameters like concentration of contaminant, dose of nZVC catalyst, pH, and reaction time on the contaminant removal efficiencies of nZVC. This review comprehensively discuss the effect of various oxidizing/reducing agents, pH of solution, synergistic effect from immobilizers/supports and various energy sources in the activation of nano zerovalent copper to produce the reactive species involved in the catalytic degradation of various organic contaminants or reductive removal of heavy metals. In the first section, the detailed discussion has been carried out on the various strategies involved in the preparation of stable nano zerovalent copper materials. A comprehensive discussion on the structure and mechanism of contaminant removal by nano zerovalent copper has been provided in the second and third section. The fourth section involved the application of nano zerovalent copper for the various organic and inorganic pollutants. The organic pollutants are further categorized into organic compounds, dye and drug molecules, and the various affecting parameters like contaminant concentration, nZVC dose, pH, time and percentage removal efficiencies are summarized in tabular form. The conclusion is mentioned at the end, with the suggestion to understand the underlying mechanism of synergistic effect for the removal of contaminants that may originate between the nano zerovalent copper and the immobilizer/support, as the future perspectives of this review article.

## 2 Synthesis of nZVC

**Figure 1:**
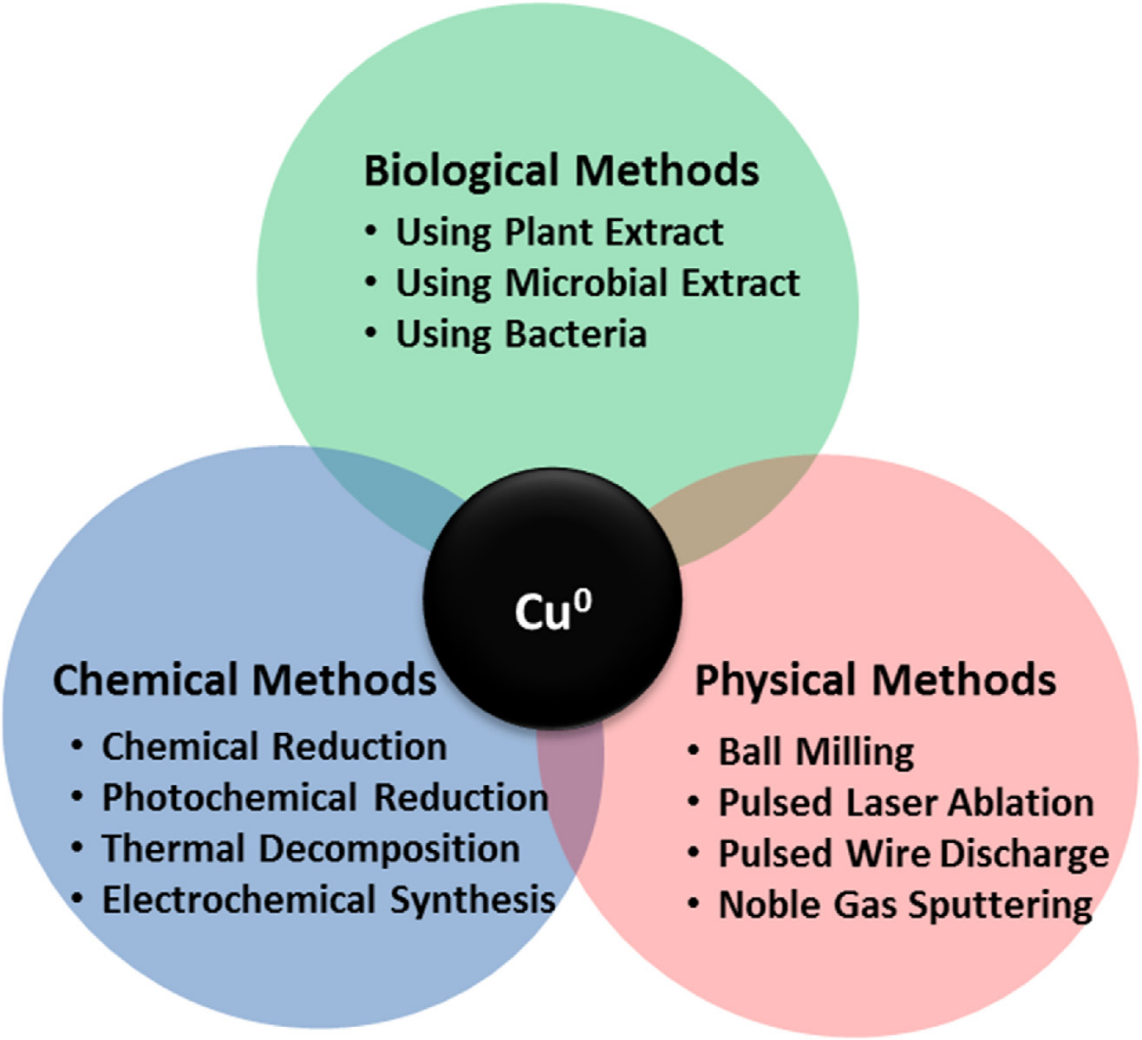
Synthetic approaches for the preparation of nZVC nanomaterial.

The catalytic efficiencies of nanoparticles as an environment remediating agent are controlled by the size of synthesized nanoparticles, material used as a support, capping material and thickness of surface oxide layer etc (Pasinszki and Krebsz, 2020). Therefore, the synthetic approach used for the manufacturing of nanoparticles plays a very significant role in deciding the properties and application of nano-catalyst obtained (Khodashenas and Ghorbani, 2014). There are generally three major categories of approaches used for the synthesis of nanoparticles namely *chemical, physical, and biological process* (Figure 1). Otherwise the different methods used for the synthesis of metallic nanoparticles includes chemical reduction (Chang et al., 2019), cathodic corrosion (Feng et al., 2018; Yanson et al., 2011), microwave-assisted (Hashimi et al., 2020), reverse-micelle (Gutierrez et al., 2021), laser-irradiation (Shahzeydi et al., 2019), electrochemical (Serra and Valles, 2018), microemulsion (Mdlovu et al., 2018), ultrasound (Sierra-Ávila et al., 2018), thermal decomposition (Numaga et al., 2020) and biogenic synthesis (El-Saadony et al., 2020) etc. The stability of synthesized copper nanoparticles is always of great concern as these can be easily oxidized on contact with air; therefore, these reactions are always performed under an inert atmosphere by purging the reaction vessel with nitrogen or argon gas. Protecting layers of organic and inorganic materials are usually employed to impart stability to nanoparticles and thus to prevent surface oxidation of copper (Begletsova et al., 2018; Kim et al., 2019; Suárez-Cerda et al., 2017). Protective agents like toluene, dodecanethiol, triethylamine, carbon, silicon, polyethylene glycols, polyacrylic acid, sodium dodecyl benzene sulfonate/sodium dodecyl sulfate, lauric acid etc. has been used in literature to prevent oxidation and thus to prepare stable copper nanoparticles (Khodashenas and Ghorbani, 2014).

### 2.1 Chemical process

#### 2.1.1 Chemical reduction process

Among the various processes used for the synthesis of metallic copper, the chemical reduction of aqueous copper salts using reducing agents such as sodium borohydride (NaBH_4_), hydrazine (N_2_H_4_), sodium hypophosphite (NaPO_2_H_2_), and ascorbic acid (vitamin C) etc. has been the most commonly employed method in literature due to its economic viability, repeatability, uniform particle size distribution and easy to perform experimental conditions.

*Reduction using NaBH*_*4*_: Theoretically speaking, the stoichiometric ratio required for the reduction reaction of Cu^2+^ using NaBH_4_ is 4:1 (Eq. 1). When the NaBH_4_ concentration dependent synthesis of Cu(0) was performed with lower NaBH_4_ concentrations, Cu_2_O and Cu(OH)_2_ were obtained as intermediates for the reduction process, which were further confirmed by XRD analysis (Liu et al., 2012). With increase in concentration of NaBH_4,_ the peak respective of Cu(OH)_2_ disappeared, however the Cu_2_O contamination was removed only when the concentration of NaBH_4_ reaches 8 times higher than that of the required stoichiometric dosage. Further, the decrease in size of copper nanoparticles was observed with increase in the dosage of NaBH_4_ for the reduction process.

1 $$4Cu^{4+}+{\mathrm{BH}}_{4}^{-}+8OH^{-}\to 4{\mathrm{Cu}}^{0}+B{\left(OH\right)}_{4}^{-}+4H_{2}O$$

*Reduction using hydrazine*: Hydrazine reduces the Cu(II) to Cu(I) at room temperature, however, at elevated temperature it converts Cu(I) to Cu(0). Nitrogen gas released during the process provides an inert atmosphere for the reduction process (Eq. 2) (Andal and Buvaneswari, 2017; Behera and Giri, 2014).

2 $${\mathrm{Cu}}^{2+}+H_{2}N-{\mathrm{NH}}_{2}\;\overset{\Delta }{\to }\;{\mathrm{Cu}}^{1+}+N_{2}+2H_{2}$$

*Reduction using sodium hypophosphite*: Sodium hypophosphite reduces Cu(II) to Cu(0) under acidic conditions in the presence of a number of capping and stabilizing agents (Eq. 3) (Lai et al., 2013; Zhu et al., 2005). Acidic condition favors the activation of sodium hypophosphite and prevents the formation of Cu(OH)_2_.

3 $$2Cu^{2+}+2H_{3}PO_{2}+2H_{2}O\;\to \;2Cu^{0}+H_{3}PO_{2}+4H^{+}$$

*Reduction using ascorbic acid*: L-Ascorbic acid not only reduces the Cu(II) to Cu(0), but also act as capping agent and provides stability to the synthesized copper nanoparticles (Eq. 4) (Umer et al., 2014).

**Figure:**
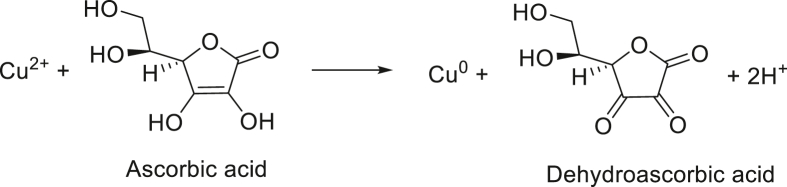

#### 2.1.2 Thermal decomposition

High temperature and pressure in a closed vessel, usually an autoclave, results in decomposition of aqueous salt of copper to copper nanoparticles. Seku et al. (2018) synthesized copper nanoparticles (average size 14 nm) with *kondagogu* a carboxymethyl gum as a capping agent using hydrothermal synthesis. Zhou et al. (2015) used supercritical hydrothermal synthesis to prepare nZVC with size ranging from 14-50 nm. Solvothermal methods have also been used for the synthesis of supported copper nanoparticles. Xu et al. (2018) used ethanol as solvent for one-pot solvothermal method for the synthesis of metallic copper doped ZnO microrods. In another solvothermal method using ethanol, Li et al. (2014) prepared copper nanoparticles supported on Li_4_Ti_5_O_12_ composites for use in Li-ion batteries. Chen et al. (2010) used facile hydrothermal method for the synthesis of metallic copper nanoparticles with morphologies like spherical, nanocubes and ribbon-like network using SDBS as stabilizer. Rahmatolahzadeh et al. (2017) used ethylenediamine and hydrazine hydrate for the facile hydrothermal synthesis of *Cu nanoparticles* with average size of 15 nm. Kumar et al. (2022) used one-pot facile hydrothermal synthesis of copper nanowires using oleylamine and oleic acid as stabilizers.

#### 2.1.3 Electrochemical synthesis

In electrochemical method of synthesis, the electric current is passed between two electrodes dipped in an aqueous solution of copper salt and sulfuric acid as an electrolyte. The synthesis of nanoparticles is observed at the electrode/electrolyte interface. Balasubramanian et al. (2017) used electrochemical deposition technique to prepare copper nanoparticles supported on reduced graphene oxide.

#### 2.1.4 Photochemical reduction

Reduction of aqueous copper ions to copper nanoparticles can be achieved using various forms of electromagnetic radiations. The intensity of light, nature of sensitizer and concentration of stabilizer/support are the determining factors for the size of copper nanoparticles. Kapoor et al. (2003) prepared copper nanoparticles under UV light using benzophenone as a photo-sensitizer and poly(N-vinylpyrrolidone) as a stabilizing agent. Wan et al. (2021) prepared ultra-small copper nanoparticles onto the surface of fullerenol using photoreduction method. Microwave is another form of electromagnetic energy with frequency range from 300 MHz to 300 GHz (Zhu et al., 2004). Kamal et al. (2019) performed microwave assisted synthesis of copper nanoparticles stabilized on carboxymethyl cellulose and bacterial cellulose as support.

### 2.2 Physical process

Physical process used for the synthesis of copper nanoparticles involves top-down approach, in which the size of bulk copper is reduced to nanoscale using various mechanical processes such as milling, pulsed laser ablation, etching, and noble gas sputtering etc.

#### 2.2.1 Ball milling method

Ball milling is the economical method used for industrial scale production of nanoparticles and is the most commonly employed physical process for conversion of millimeter sized copper fillings to nanosized copper using stirred ball mills. Capping agents are added during the milling process to prevent the oxidation of copper nanoparticles. Further, the milling speed, temperature, time, type of vessel, atmosphere, and stabilizing agent decides the size and properties of the prepared copper nanoparticles.

#### 2.2.2 Pulsed laser ablation

Pulsed laser ablation technique involves the degradation of a solid copper in an inert atmosphere in a closed chamber under vacuum upon irradiation with a high energy laser beam to form plasma, which on cooling in the presence of solvent give a colloidal solution (Sadrolhosseini et al., 2019). The quality of the nanoparticles is influenced by the type of solvents used for the ablation, intensity of laser beam and duration of pulsing etc. Tyurnina et al. (2014) used pulsed laser irradiation (wavelength - 1064 nm, duration - 100 ns) for the synthesis of stable colloidal solution of copper nanoparticles. Goncharova et al. (2019) also used pulsed laser radiation for a duration of 7 ns for the synthesis of copper nanoparticles and studied the role of different solvent systems like ethyl alcohol, NaOH and H_2_O_2_ in deciding the morphology of the prepared nanoparticles.

#### 2.2.3 Pulsed wire discharge method

This method is applied to metals with high ductility and electrical conductivity. Due to its high cost and effectiveness for fewer metals, this method has limited industrial applications for the synthesis of nanomaterial. Tokoi et al. (2013) synthesized copper nanoparticles from copper wire in deionized water by applying power ranging from 0.8 to 5.5 kV. Murai et al. (2007) prepared copper nanoparticles (size 10–25 nm) with organic coating via evaporation of copper by wire explosion method in oleic acid mist. In another method *Cu nanoparticles* of size 16–43 nm were synthesized by applying power of 22 kV under a pressure of 0.1 MPa (Dash and Balto, 2011).

#### 2.2.4 Noble Gas Sputtering

Inert-gas condensation method has been used to prepare copper nanoparticles based on DC magnetron sputtering using noble gases like Ar or He. Soganci et al. (2018) prepared copper nanoparticle decorated graphene using noble gas sputtering technique with average particle size of 5 nm. Dong et al. (2018) decorated TiO_2_ nanotube arrays with *Cu nanoparticles* through magnetron sputtering using Ar gas under pressure (10 mTorr).

### 2.3 Biological process

**Figure 2:**
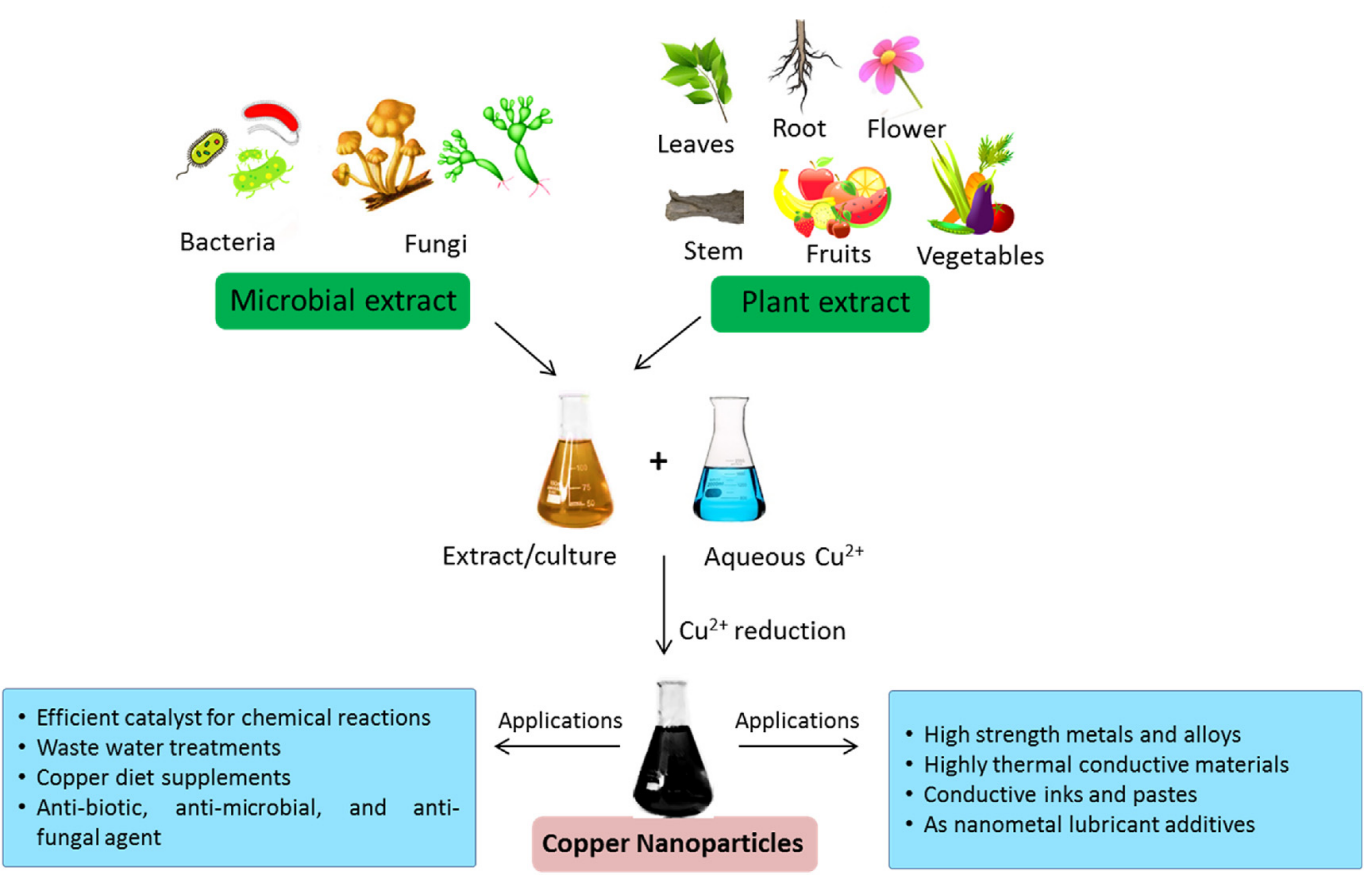
Green synthesis of copper nanoparticle.

Other than chemical and physical processes, the green method of obtaining metallic copper has also been successfully explored owing to their low cost and eco-friendly nature bearing no adverse impacts on the environment (Suresh et al., 2013) (Figure 2). The green method involves the use of plant extracts or metabolic activities of microorganisms like bacteria and fungi for the reduction of Cu^II^ to Cu^0^ (Thiruvengadam et al., 2019).

#### 2.3.1 Using plant extract

Bio-extracts obtained from various plant parts (leave, stem, bark, root etc.) includes metabolites such as alkaloids, flavonoids, terpenoids, polyphenols, proteins etc., not only acts as reducing agent, but also stabilizes the produced nanoparticle by providing a protective capping onto it. Different bio-extracts used for the synthesis of copper nanoparticles includes *Eucalyptus camaldulensis* (river red gum) (Asghar and Asghar, 2020)*, Murraya koenigii* (curry tree) (Pantawane et al., 2020; Pragyan and Pragnya, 2019)*, Azadirachta indica* (neem) (Abhiman et al., 2018; Nagar and Devra, 2018)*, Avicennia marina* (grey mangrove) (Essa et al., 2021)*, Datura stramonium* (jimsonweed) (Parikh et al., 2014)*, Rosa rubiginosa* (sweet briar) (Asghar and Asghar, 2020)*, Magnolia kobus* (Lee et al., 2013), *Cissus arnotiana* (Rajeshkumar et al., 2019), *Terminalia arjuna* (arjun tree) (Sharma and Gupta, 2021; Yallappa et al., 2013)*, Citrus limon* (lemon) (Amer and Awwad, 2021)*, Syzygium aromaticum* (Clove) (Rajesh et al., 2018), *Eclipta prostrata* (false daisy) (Chung et al., 2017)*, Ginkgo biloba* (Nasrollahzadeh and Sajadi, 2015), *Punica granatum* (pomegranate) (Padma et al., 2018) etc.

#### 2.3.2 Using microbial extract

Extracts from cultured microorganisms like fungi and bacteria have been used for the synthesis of metallic copper (Varshney et al., 2010).

#### 2.3.3 Using bacteria

Prokaryotes such as bacteria owing to their ease of culture, high multiplication rate and optimum culture condition has been used for biogenic synthesis of copper nanoparticles (Shobha et al., 2014). *Morganella psychrotolerans* and *Morganella morganii* RP42 were used for the synthesis of nZVC with particle size in the range of 15–20 nm (Ramanathan et al., 2011, 2013). Varshney et al. (2010) employed a rapid biological process to synthesize copper nanoparticles (size 8–15 nm) using *Pseudomonas stutzeri*, a non-pathogenic bacterium. In another study, Varshney et al. (2011) also utilized *Pseudomonas stutzeri* to synthesize cubic shaped copper nanoparticles (size 50–150 nm) using wastewater from electroplating process.

#### 2.3.4 Using algae and fungi

Fungi secrete a large number of enzymes such as NADH and NADPH during their metabolic processes, and thus play a significant role in the biosynthesis of copper nanoparticles (Noor et al., 2020). The different algal and fungal species used for the synthesis of *Cu nanoparticles* includes *Penicillium aurantiogriseum*, *Penicillium waksmanii* and *Penicillium citrinum* (Honary et al., 2012), *Fusarium oxysporum* (Majumder, 2012), *Hypocrea lixii* (Salvadori et al., 2013), *Bifurcaria Bifurcata* (Abboud et al., 2014), *Trichoderma koningiopsis* (Salvadori et al., 2014), *Chlamydomonas reinhardtii* (Žvab et al., 2021), *Botryococcus braunii* (Arya et al., 2018) etc.

## 3 Structure of nZVC

The typical structure of nZVC prepared using a bottom-up approach consists of a core-shell structure having zerovalent metallic copper as the core and an oxide shell consisting of Cu_2_O and CuO (Kim et al., 2013; Liu et al., 2019). The surface oxidation is inevitable due to the pyrophoric nature of copper nanoparticles, until they are synthesized and stored in an inert atmosphere (Alymov et al., 2018). The nZVC core acts as the powerhouse of electrons. The transfer of charge from core through intermediate semiconducting copper oxide layer causes reductive degradation of contaminants on the surface of core-shell nanoparticles (Fathima et al., 2018; Shikha et al., 2015). Stabilizers and capping agents prevent the surface oxidation and thus render extra stability to the nano zerovalent copper (Din and Rehan, 2017). Protective agents like toluene, dodecanethiol, triethylamine, carbon, silicon, polyethylene glycols, polyacrylic acid, sodium dodecyl benzene sulfonate/sodium dodecyl sulfate, lauric acid etc. has been used in literature to prevent oxidation and thus to prepare stable copper nanoparticles (Khodashenas and Ghorbani, 2014). Kim et al. (2013) prevents the surface oxidation of nZVC through the formation of copper–copper formate core–shell nanoparticles. Organic/inorganic support employed for the immobilization provides extra stability to the synthesized nZVC via minimization of aggregation and leaching of Cu(I/II) under unfavorable conditions. The various organic/inorganic materials used as support for the synthesis of nZVC includes zeolite (Danish et al., 2017), Fe_2_O_3_ (Kim and Ko, 2018), green rust (Fang et al., 2019), ZrSiO_4_ (Mahmoud et al., 2021), microscale zinc (Li et al., 2020), chitosan (Wu et al., 2009), TiO_2_ (Alani et al., 2021), cellulose filter paper (Kamal et al., 2016), carboxymethyl cellulose (Kamal et al., 2019), carbon nanotubes (Zheng et al., 2021), biochar (Din et al., 2021), graphene (Chi et al., 2019; Xu et al., 2019), montmorillonite (Hong et al., 2017), pistachio shell powder (Kumar et al., 2021) etc. The experimental conditions and the amount of the supporting materials decide the size and surface area of the synthesized zerovalent copper nanoparticles.

## 4 Mechanism of contaminant removal

**Figure 3:**
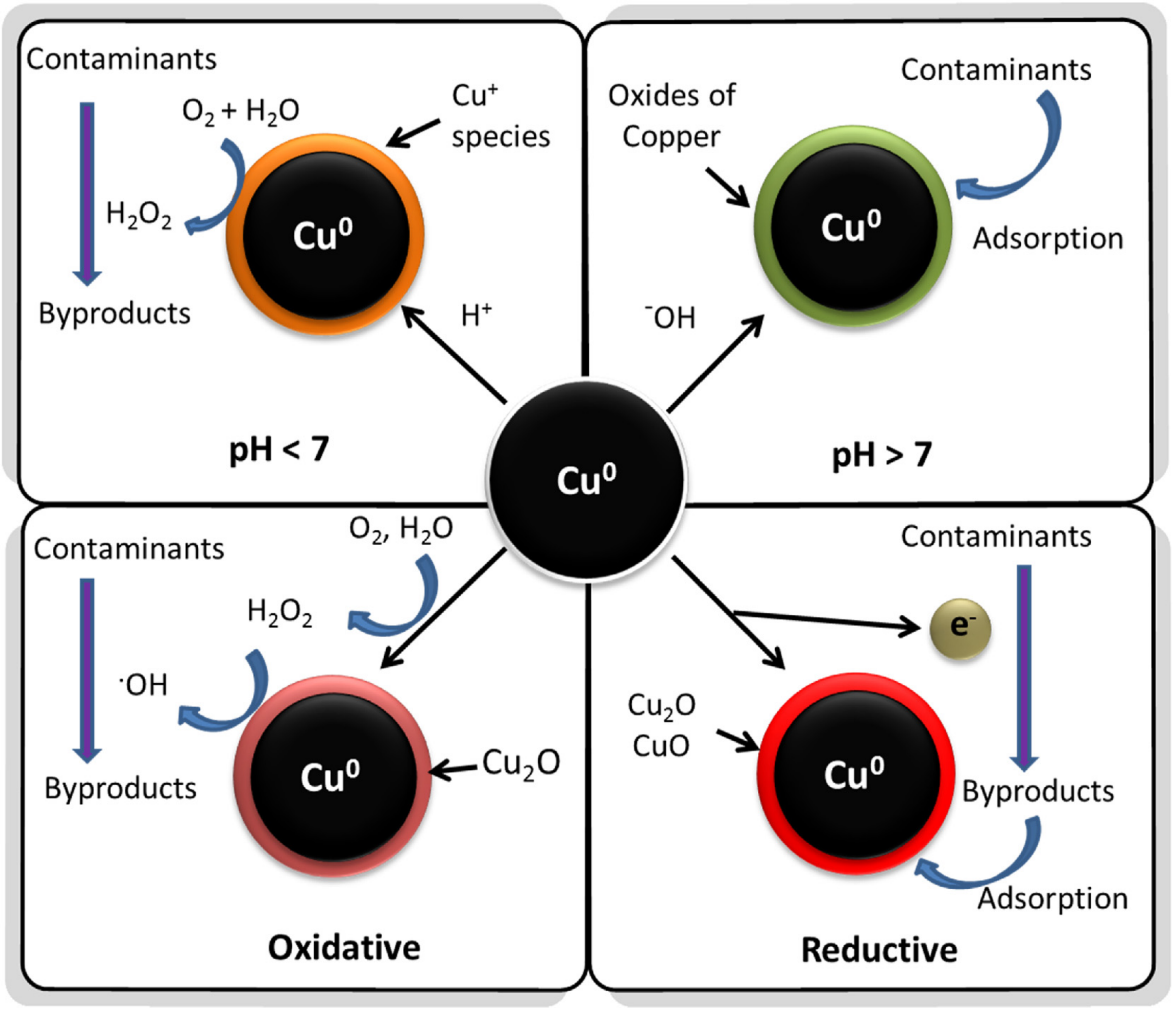
Schematic of various mechanisms involved in nZVC mediated contaminant removal processes.

Depending on the studies performed to identify the species of copper involved in the removal of various organic/inorganic contaminants, it has been observed that there are mainly three removal processes whereby the copper nanoparticles has been involved in environment remediation phenomenon namely reductive degradation, oxidative removal, and surface adsorption (de Sousa et al., 2019a). In the absence of dissolved oxygen (anoxic), the removal process primarily involves the transfer of electrons from Cu^0^, resulting in reductive degradation/removal of organic/inorganic contaminants. Further, the stability of Cu^0^ remains intact in the absence of oxygen owing to suppression of hydroxyl radical formation. Conversely, the presence of oxygen (oxic) generates various reactive oxygen species (ROS) like H_2_O_2_, ^1^O_2_, O_2_^.-^, ^**.**^OH etc. via transfer of electrons from Cu^0^, which further causes oxidative degradation of organic contaminants (Wen et al., 2014) (Figure 3). Different radical scavengers have been studied to find the key radical species involved in the degradation mechanisms. In studies, tert-butyl alcohol (TBA) inhibits hydroxyl radicals (^**.**^OH) (Qi et al., 2016; Wang et al., 2019), superoxide dismutase (SOD) inhibits superoxide anions (O_2_^.-^) (Dror et al., 2020), sodium azide (NaN_3_) inhibits singlet oxygen (^1^O_2_) (Gao et al., 2021), catalase (CAT) was used to identify hydrogen peroxide H_2_O_2_ (Shen et al., 2017), combination of isopropanol with TBA was used to ascertain the type of hydroxyl radical in degradation process (Ma et al., 2018) etc.

The pH of the reaction mixture contributes significantly in deciding the mechanism of the contaminant removal process. At lower pH (less than 7), the Cu^+^ species predominates in the solution, which promotes *in-situ* generation of H_2_O_2_ from dissolved O_2_ and H_2_O in a Fenton-like process, and later on also participate in the decomposition of H_2_O_2_ to ^.^OH radicals, thus controlling the overall degradation process (Xu et al., 2019). At higher pH (more than 7), the removal of contaminants via surface adsorption prevails due to the formation of copper oxides (CuO) and copper hydroxides (Cu(OH_2_) on the surface of copper nanoparticles (Ourique et al., 2018). External oxidizing agents like hydrogen peroxide (Ma et al., 2018), peroxymonosulfate (Zhou et al., 2016) etc. and reducing agents like sodium borohydride (Raut et al., 2016) etc. have also been studied with zerovalent copper for the various degradation processes.

## 5 Applications of nZVC for environment remediation

### 5.1 Removal of organic contaminants

Organic contaminants or waste are the compounds comprising carbon, hydrogen along with other heteroatoms. These contaminants depending on their physical nature may exist in the environment as gas or liquid phase (volatile organic compounds, VOCs) or in solid phase (chemicals, waxes, plastics, resins etc.). Because of their unwanted toxicity associated with their complex structure, they always pose a great threat to flora and fauna. Researchers across the world are working restlessly to develop new methods and state of art techniques to find a solution to this problem. Tailored nanoparticle mediated degradation of organic contaminants provide a sustainable and long term solution. Zerovalent copper is one such promising material used in literature for the remediation of organic and inorganic contaminants. So this part of the review will focus on the reports discussing the application of zerovalent copper for the removal of organic contaminants (Table 1).

**Table 1:** nZVC mediated removal of organic contaminants.

nZVC (bare or supported)	Pollutant	Oxidizing /Reducing agent (Conc.)	Optimum experimental conditions				Removal (%)	Ref.
			nZVC dose	Pollutant conc.	pH	Time		
Bare	Dichloromethane	NaBH_4_ (1 g^.^L^−1^)	2.5g^.^L^−1^	26.4 mg^.^L^−1^	-	1h	90%	(Huang et al., 2012)
Reduced B_12_	Dichloromethane	-	0.1 g^.^L^−1^	26 mg^.^L^−1^	-	2h	99%	(Huang et al., 2013)
Bare	2,4-Dichlorophenol	-	2 g^.^L^−1^	7.5 mg^.^L^−1^	6.2	5 days	67.1%	(Chang et al., 2019)
Bare	Monochloro-aromatics	NaBH_4_ (1.0 g.L^−1^)	2.5 g^.^L^−1^	34 mg.L^−1^	9.5	12h	100 %	(Raut et al., 2016)
Bare	1,2-Dichloroethane	NaBH_4_ (0.95 g^.^L^−1^)	2.5 g^.^L^−1^	30 mg^.^L^−1^	-	2h	80%	(Huang et al., 2011)
Green rust	Tetrabromo-bisphenol A	-	0.5 g^.^L^−1^	15 mg^.^L^−1^	8	5h	92.11%	(Fang et al., 2019)
Zeolitic imidazolate framework	p-Nitrophenol	NaBH_4_ (6.6 g.L^−1^)	1.5 g^.^L^−1^	25 mg^.^L^−1^	-	2 min.	100 %	(Yang et al., 2019)
Bare	Benzoic acid	PMS (1 mM)	40 mg^.^L^−1^	4.9 mg.L^−1^	3	10 min.	100 %	(Zhou et al., 2016)
Zeolite	Trichloroethylene	SPC (47.1 g^.^L^−1^)	0.2 g^.^L^−1^	19.7 mg^.^L^−1^	-	2h	>95%	(Danish et al., 2017)
Bare	Diethyl phthalate	-	0.5 g^.^L^−1^	10 μM	2.5	2h	100%	(Wen et al., 2014)
Bare	4-Chlorophenol	-	100 g^.^L^−1^	40 mg^.^L^−1^	3	4h	65%	(Duan et al., 2016)
Bare	aniline	O_3_ (10 mg^.^L^−1^)	2 g^.^L^−1^	10 mg.L^−1^	6	24 min.	98%	(Zhang et al., 2017a)
Bare	Bisphenol AF	PS (0.27 g^.^L^−1^)	0.5 g^.^L^−1^	6.7 mg^.^L^−1^	4.0	20 min.	97.0%	(Wang et al., 2019)
Bare	Ether amine surfactant	Ascorbic acid (88.1 g^.^L^−1^)	60 mg^.^L^−1^	180 mg^.^L^−1^	6	4 h	57%	(Martins et al., 2021)
ZrSiO_4_@NPANI	Nitroanilines	NaBH_4_ (1 g^.^L^−1^)	300 μL	27.6–69 mg^.^L^−1^	7	6–9 min.	98.4%	(Mahmoud et al., 2021)
Microscale zinc	4-Nitrophenol	-	2.34 g^.^L^−1^	6.6 mg^.^L^−1^	2.5	60 min.	77%	(Li et al., 2020)

#### 5.1.1 Chlorinated organic compounds (COCs) as pollutants

Chlorinated organic compounds (COCs) are the most common terrestrial contaminants having anthropogenic origin and used for different applications, such as degreasing, dry cleaning, and pesticides. Due to their toxic and persistent nature, these cause a major threat to the environment.

Dichloromethane (DCM) **1** is an industrial origin organic contaminant known for its high carcinogenicity and hepatotoxic effects. Zerovalent metallic nanoparticles are known for the treatment of chlorinated organic contaminants (COCs) such as tri- and tetrachloromethane, but are found less effective for dichloromethane and dichloroethane probably due to their relatively stronger carbon-chlorine bonds compared to higher-chlorinated organic compounds (Doong et al., 2003; Feng et al., 2005; Wang et al., 2009). However, zerovalent copper (ZVC) was observed as an effective catalyst for dechlorination reactions of dichloromethane under NaBH_4_ reduction conditions owing to its small size (50 nm) and high surface area (19 m^2^ g^−1^) (Huang et al., 2012). Under optimized conditions with 2.5 g^.^L^−1^ of ZVC and 1 g^.^L^−1^ of NaBH_4_, nearly 90% of DCM (26.4 mg^.^L^−1^) was degraded within 1 h of reaction time. Chloride ions were the immediate product of the degradation reaction as indicated by IC analysis of the reaction mixture. The pseudo-first-order rate constant value (2.19 h) was found to be 2–3 times higher for the ZVC mediated dechlorination of DCM as compared to the other zerovalent metals. Further, the concentration of the leached copper ions in solution was within the permissible limit (2 mg^.^L^−1^) for drinking water as per WHO standard. Lin et al. (2005) used cation resin supported zerovalent copper nanocomposite for the effective removal of carbon tetrachloride (CCl_4_) from wastewater. Cation resin not only prevents the agglomeration of ZVC particles, but also adsorbs the CCl_4_ on its surface to facilitate the removal process. Further, the exchange between Cu^2+^ and H^+^/Na^+^ on the resin, keeps the solution pH between 3 and 4, favorable for the CCl_4_ dechlorination and Cu^2+^ concentrations less than 0.1 mg^.^L^−1^.

Vinyl chloride monomer (VMC) is used for the synthesis of polyvinyl chlorides (PVC), a versatile material with several industrial applications (Endo, 2002). 1,2-Dichloroethane (1,2-DCA) **2** is used for the synthesis of vinyl chloride (Liang et al., 2019) and usually the groundwater nearby the VCM industrial plants has been found contaminated with 1,2-DCA (Wang et al., 2015). Huang et al. (2011) used zerovalent copper nanoparticles for the effective remediation of 1,2-DCA using NaBH_4_ as electron donor. The 1,2-DCA (30 mg^.^L^−1^) degradation efficiency of more than 80% was observed within 2 h using optimum ZVC dose of 2.5 g^.^L^−1^ and borohydride concentration of 25 mM. Measured oxidation-reduction potential revealed higher negative values (−1100 eV) with increasing NaBH_4_ concentrations indicating strong reducing conditions prevailed during reaction. When used individually, neither ZVC nor NaBH_4_ could degrade/reduce 1,2-DCA. Formation of ethane as the major product (79%) of the 1,2-DCA degradation suggests ZVC mediated two successive hydrogenolysis reactions as a major reaction pathway. The formation of ethylene as the minor product (1%) indicates the presence of dihaloelimination also as the part of degradation reaction.

Huang et al. (2013) evaluated the Cu–B_12_ system for its synergistic effect for the degradation of DCM using Tri-citrate as a reducing agent. The ZVC acts as electron donor and vitamin B_12_ performs the role of electron mediator in the overall reductive degradation process of DCM. Batch experiments performed to study the degradation process showed that nearly 99% of the DCM (26 mg^.^L^−1^) was degraded reductively to give methane and methyl chloride as products within 2 h of reaction time by Cu –B_12_ system, which was significantly higher than that of using B_12_ alone. The rate of DCM degradation was observed to be a function of ZVC dose and increases rapidly with increase in ZVC concentration (<0.1 g^.^L^−1^). Tri-citrate mediated reduced form of B_12_ was responsible for the DCM degradation on the surface of ZVC. The pseudo first order rate of reaction was observed with a rate constant of 1.35 h^−1^, which was higher (5 times) than that of vitamin B_12_ when used alone.

Chang et al. (2019) investigated the degradation efficiencies of zerovalent copper nanoparticles (Cu-GT NPs) synthesized via green approach using green tea extract towards the organic contaminant 2,4-dichlorophenol (2,4-DCP) **3** and a comparison was performed with the chemically synthesized zerovalent copper (Cu-SB) nanoparticles for their physico-chemical properties. The particle sizes of 5 nm and 200 nm were determined using dynamic light scattering (DLS) technique for Cu-GT NPs and Cu-SB, respectively. The Cu-SB nanoparticles were observed to possess higher reducibility and reactivity than Cu-GT NPs. Higher reductive degradation efficiencies (67.1%) of 2,4-DCP (7.5 ppm) observed with Cu-SB (2 g^.^L^−1^) at near neutral pH (6.2) with incubation period of 5 days instead of its lower dispersion and stability compared to Cu-GT NPs was ascribed to the higher activation energy of Cu-SB particles (29.65 kJ mol^−1^) for 2,4-DCP degradation. Further, the lower reactivity of Cu-GT NPs was associated with formation of a high percentage of monovalent copper. Lee et al. (2010) compared the nZVC mediated degradation of chlorobenzene (Cl–B) **4** in the presence and absence of microwave (MW) energy. The MW energy directly generates the heat inside the nZVCs that causes an increase in CB degradation efficiency (1.8 times) from 19.5% to 41.3% by decreasing the activation energy from 21.4 kJ mol^−1^ to 15.8 kJ mol^−1^.

Raut et al. (2016) evaluated the degradation efficiencies of zerovalent copper nanoparticles synthesized via chemical reduction using sodium borohydride for the monochloro-aromatic compounds such as chlorobenzene (Cl–B) **4**, chloropyridine (Cl-Py) **5**, chlorotoluene (Cl-T) **6** and chlorobiphenyl (Cl-BPh) **7**. Under the optimized conditions using ZVC nanoparticles (2.5 g^.^L^−1^) and NaBH_4_ (1.0 g^.^L^−1^) as reducing agents, nearly 100% dechlorination was observed for all chloro-aromatics within 12 h. GCMS and NMR techniques were used to analyze the reaction intermediate and dechlorinated products to determine the reaction pathway. Acidified isopropyl alcohol was also used as a reducing agent with ZVC nanoparticles, but displayed lower degradation efficiency of 70% only. Further, higher oxidation-reduction potential observed for NaBH_4_ (−1016 mV) in comparison to acidified alcohol (−670 mV) under reaction conditions, confirmed the effectiveness of ZVC-NaBH_4_ for reductive degradation of chloro-aromatics.

Zeolite supported nano zerovalent iron-copper bimetallic composite (Z-nZVFeCu) synthesized by ion exchange method was used to investigate degradation efficiency of trichloroethylene (TCE) via sodium percarbonate (SPC) activation (Danish et al., 2017). The Z-nZVFeCu acts as a heterogeneous Fenton like catalyst to degrade TCE (> 95%) through the formation of hydroxyl radicals in the system. Higher concentration of hydroxyl radicals were generated using Z-nZVFeCu system as compared to Z-nZVFe (without ZVC) and nZVFe (without zeolite support and ZVC) as analyzed by benzoic acid, a probe indicator used for quantification of ^**.**^OH radicals. The enhanced catalytic efficiency of Z-nZVFeCu compared to Z-nZVFe and nZVFe was ascribed to its better stability due to fewer leaching of Cu and Fe from Z-nZVFeCu system, higher surface area of adsorption for rapid diffusion of reactant and product and increased surface active sites resulted from dispersed Fe–Cu bimetallic nanoparticles on the surface of natural zeolite.

#### 5.1.2 Phenolic organic pollutants

Phenolic compounds are persistent organic pollutants and due to their long term effect on human health, these are enlisted by the European Union (EU) and United States Environmental Protection Agency (USEPA) as pollutants of priority concern (Mahugo-Santana et al., 2010).

**Figure 4:**
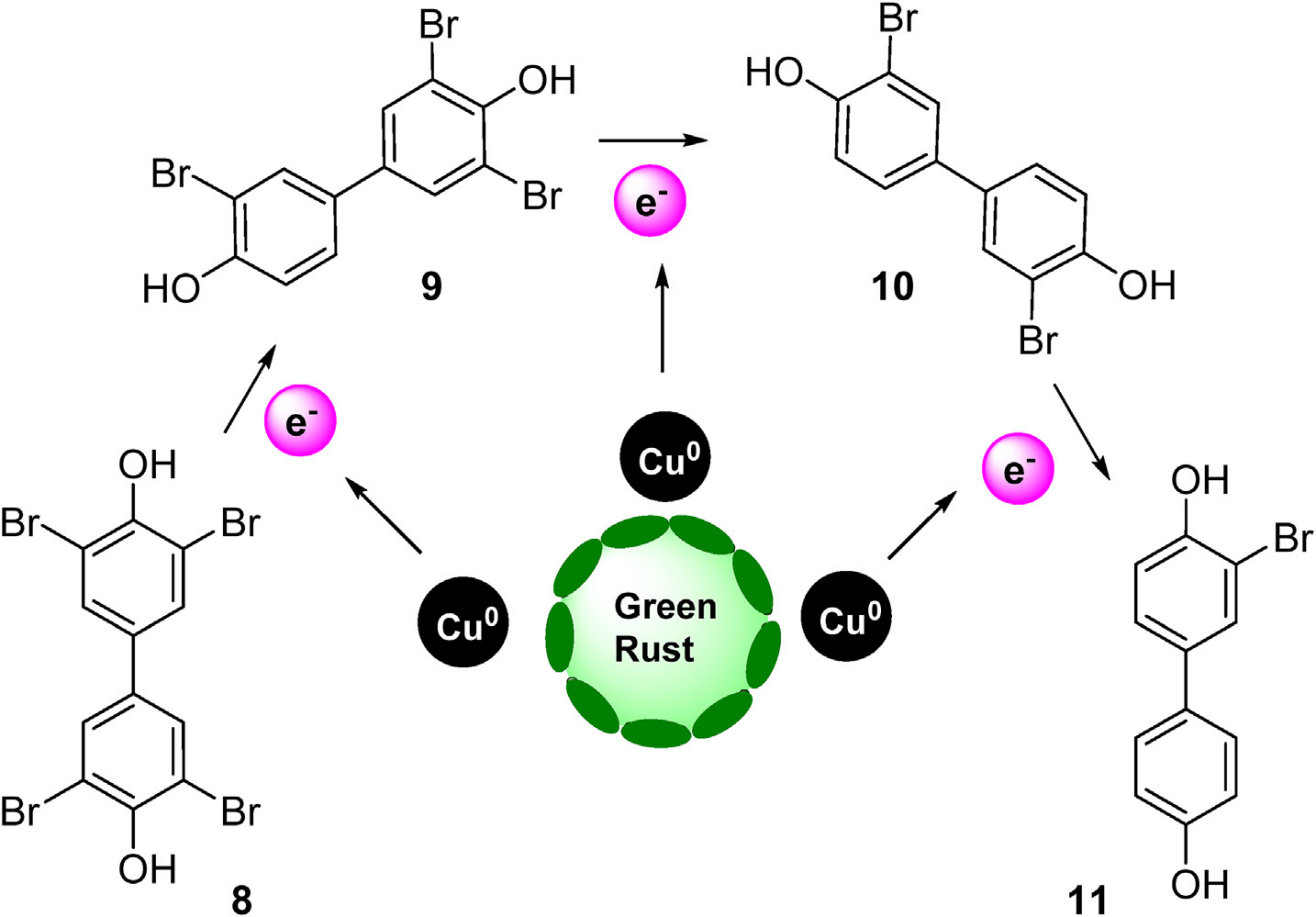
Proposed GR(Cl)–Cu NPs mediated reductive debromination pathway for removal of TBBPA **8** (drawn from Fang et al., 2019).

Tetrabromobisphenol A (TBBPA) **8** is the most commonly employed brominated flame retardant (Covaci et al., 2009). However, it is considered as a very harmful contaminant due to its high neurotoxicity, cytotoxicity and immune toxicity associated with its structural features (Lai et al., 2015; Yu et al., 2019). Green rust (GRs) are obtained by partial oxidation of Fe(II) or reduction of Fe(III) salts (Bhave and Shejwalkar, 2018). Green rusts (GRs) interlayered with Cl^−^, CO_3_^2-^, and SO_4_^2-^, were effectively used for the dehalogenation of TBBPA (Fang et al., 2019) (Figure 4). However, the zerovalent copper modified green rust with interlayered Cl^−^ ion exhibits enhanced reductive reactivity for TBBPA. The presence of CO_3_^2-^ and SO_4_^2-^ has a negative effect on the TBBPA reduction by GR(Cl)–Cu NPs and GR(Cl). The Galvanic cell model was explained for the GR(Cl)–Cu NPs mediated TBBPA reduction, where the electrons were transferred from GR(Cl) to Cu NPs for TBBPA reduction. The reaction intermediates of TBBPA reductive debromination were investigated using LC-MS and were identified as monobromobisphenol A (Mono-BBPA) **11**, dibromobisphenol A (Di-BBPA) **10**, and tribromobisphenol A (Tri-BBPA) **9**.

Nanocomposite ZVC/ZIF-8, a hybrid of zerovalent copper (ZVC) and zeolitic imidazolate framework (ZIF) was obtained by Yang et al. (2019) via immobilizing ZVC (2–8%) onto the surface of ZIF by single-step chemical reduction method. This integrated ZVC/ZIF-8 catalyst exhibits good catalytic efficiency for the p-nitrophenol (PNP) **12** reduction. The complete PNP (0.18 mM) reduction was observed in the presence of NaBH_4_ (0.17 M) within 2 min of reaction time, using 6%-ZVC/ZIF-8 hybrid (0.003 mg) with highest observed catalytic efficiency and rate constant value of 2.39 min^−1^. In another study, Li et al. (2020) prepared mZn/Cu bimetallic particles by depositing ZVC on surface of Zn and compared it for the p-nitrophenol degradation analysis with other microscale particles like copper on iron (mFe/Cu), metallic iron (mFe), metallic zinc (mZn), metallic copper (mCu), and metallic zinc + metallic copper (mZn + mCu). The removal of total organic carbon (TOC) was observed maximum with mZn/Cu (77%) compared to other systems studied i.e. mZn (5%), mCu/Fe (41%), mCu (19%), mFe (7%), and mZn + mCu (9%). The mZn/Cu system also produced the maximum mineralization, suggesting the oxidative degradation of PNP by ^**.**^OH radicals from Fenton-like reactions.

Bisphenol A (2,2-bis(4-hydroxyphenyl)propane, BPA) **14** is an industrial chemical used in the manufacture of epoxy resins and polycarbonate plastics (Abraham and Chakraborty, 2020). However, the BPA is regarded as an endocrine disrupting chemical (Matsushima et al., 2021) and is associated with many adverse effects in humans, including birth defects, development disorders, cancerous tumor, reduced immunity and decreased semen quality etc (Kapustka et al., 2020; Rebai et al., 2021). Organic molecules functionalized ZVC nanoparticles (CuHT, CuPET, and CuPA) synthesized via borohydride reduction of copper(II)nitrate trihydrate salt in the presence of hexanethiol (HT), phenylethanethiol (PET), and phenylacetylene (PA) exhibited significant electro-catalytic activity for oxidation of BPA (Guo et al., 2019). The different pH dependent mechanisms were proposed for BPA oxidation. At pH < 7, ZVC mediated BPA decomposition involves the formation of phenol radical along with isopropyl phenol, which further undergoes one proton oxidation process. However at pH > 7, monohydroxylated BPA formed required two protons for further oxidation. The difference in catalytic efficiencies of *Cu nanoparticle*-modified electrodes was ascribed to outer layer ligands and the proton environment for the BPA oxidation.

**Figure 5:**
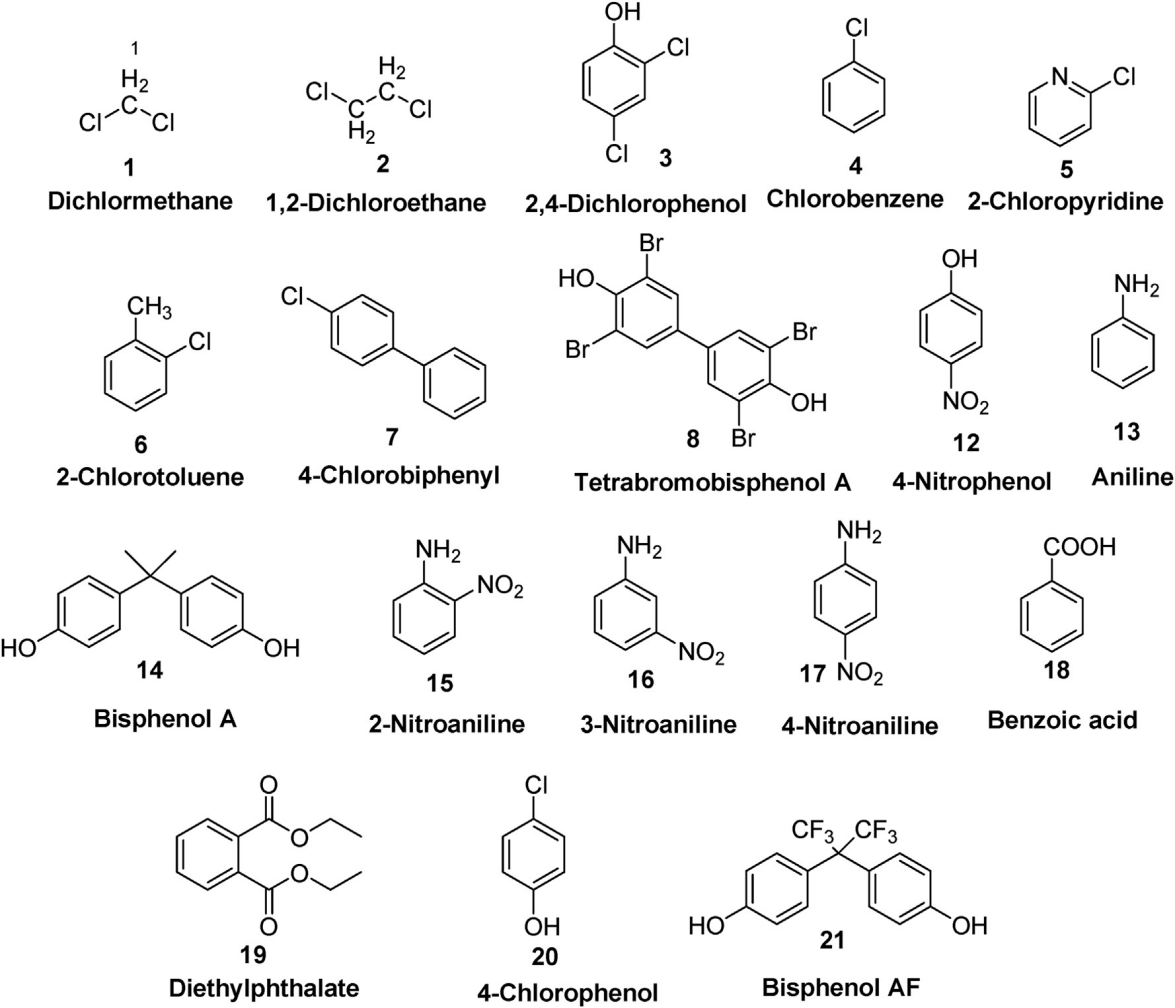
Organic molecules studied for the nZVC catalyzed removal processes.

In another study performed by Wang et al. (2019b) zerovalent copper mediated activation of persulfate in conjugation with ultrasound radiation was used to investigate bisphenol AF (BPAF) **21** degradation under acidic conditions. The BPAF degradation rate was synergically enhanced from 59.8% to 97.0%, while coupling the heterogeneous Fenton process with the sonolysis. The optimized conditions for the BPAF removal process includes BPAF conc. of 20 mol L^−1^, ZVC conc. of 0.5 g^.^L^−1^, PS conc. of 1 mM and ultrasound energy of 120 W at 20 kHz (Figure 5).

Duan et al. (2016) investigated the ZVC mediated reductive dechlorination of 4-chlorophenol (4-CP) **20** in aqueous medium and comparison was made with the Cu–Fe bimetallic nanocomposite material. ZVC exhibits higher 4-CP dechlorination efficiency (65%) via direct electron transfer reduction with formation of cyclohexanone as product, whereas the Cu–Fe bimetallic system causes dechlorination via indirect hydrogenation with formation of phenol. The observed dechlorination efficiency of ZVC was a result of conjugation between orbitals of Cu^0^ and π-orbitals of the benzene of 4-CP. Dechlorination of aliphatic chlorinated compounds such as mono- and dichloroacetic acids was not observed with ZVC due to lack of conjugation.

#### 5.1.3 Aromatic amines as pollutants

Aromatic amines are used as source or intermediates in the synthesis of a number of organic compounds of industrial importance such as dyes, drugs, pesticides, fertilizers, cosmetics, polymers, surfactants etc. Due to their carcinogenic nature and bioaccumulations, these are considered an important class of anthropogenic contaminant (Ferraz et al., 2012).

Among the various zero-valent metals (Fe^0^, Co^0^, Al^0^, Cu^0^) studied for the ozonation degradation of aniline **13**, Cu^0^ was observed to be the best for the catalytic ozonation activity (Zhang et al., 2017). A significant destruction (98%) of aniline (10 mg^.^L^−1^) via ozone activation was observed with ZVC (2 g^.^L^−1^) in a pH range of 4–10 within 24 min of reaction time. EPR analysis confirmed that OH radical was the active species responsible for the aniline degradation, formed from Fenton-like reactions between Cu^+^ and H_2_O_2_, which were further resulted from a series of redox reactions between ZVC and O_3_.

Nitroanilines are considered highly toxic and methods/techniques have extensively been developed for their removal or transformation to a less toxic form in the aqueous medium. In one of such approach, Mahmoud et al. (2021) designed a zirconium silicate-nanopolyaniline supported nano zerovalent copper nanocatalyst to perform the catalytic reduction studies of nitroanilines such as 2-nitroaniline **15**, 3-nitroaniline **16**, and 4-nitroaniline **17**. The pseudo-first order kinetics were followed with observed rate constants (*k*) values 0.188, 0.246 and 0.114 min^−1^ for 2-nitroaniline, 3-nitroaniline, and 4-nitroaniline, and the nZVC on the nanocatalyst was considered as the center for the electron transfer process to nitroanilines for the NaBH_4_ mediated reduction.

#### 5.1.4 Aromatic acids and its derivatives as pollutants

Zhou et al. (2016) evaluated the benzoic acid (BA) **18** degradation by using nano-zerovalent copper (nZVC) catalyzed activation of peroxymonosulfate (PMS) under acidic conditions. Complete degradation of BA was achieved with the nZVC/PMS process at initial pH 3 within 10 min of reaction time. Acidic catalyzed release of Cu(I) from ZVC generates hydroxyl radicals (^**.**^OH) from Fenton like process and sulfate radicals (SO_4_^**.-**^) from PMS activation process were responsible for the increased BA degradation rates. The pseudo-first-order kinetics was observed for the BA degradation, with rate constant value of 0.355 min^−1^.

Wen et al. (2014) performed the oxidative degradation studies of Diethyl phthalate (DEP) **19** using zerovalent copper (ZVC) under oxic conditions. Diethyl phthalate was completely degraded within 2 h of reaction time using 0.5 g^.^L^−1^ of ZVC at initial pH 2.5. Under acidic conditions, Cu^+^/Cu^2+^ redox coupled Fenton-like process results in formation of H_2_O_2_ from O_2_, followed by its decomposition to ^**.**^OH radicals was responsible for degradation of DEP, which was further confirmed by inhibition of DEP degradation in the presence of *tert*-butanol, a ^**.**^OH radical scavenger.

Martins et al. (2021) obtained ZVC nanoparticles via recycling process of printed circuit of lead-free computer motherboards and employed as Fenton-like catalyst in the presence of ascorbic acid for the degradation studies of ether amine surfactant used in mining floating.

### 5.2 Removal of dyes

**Figure 6:**
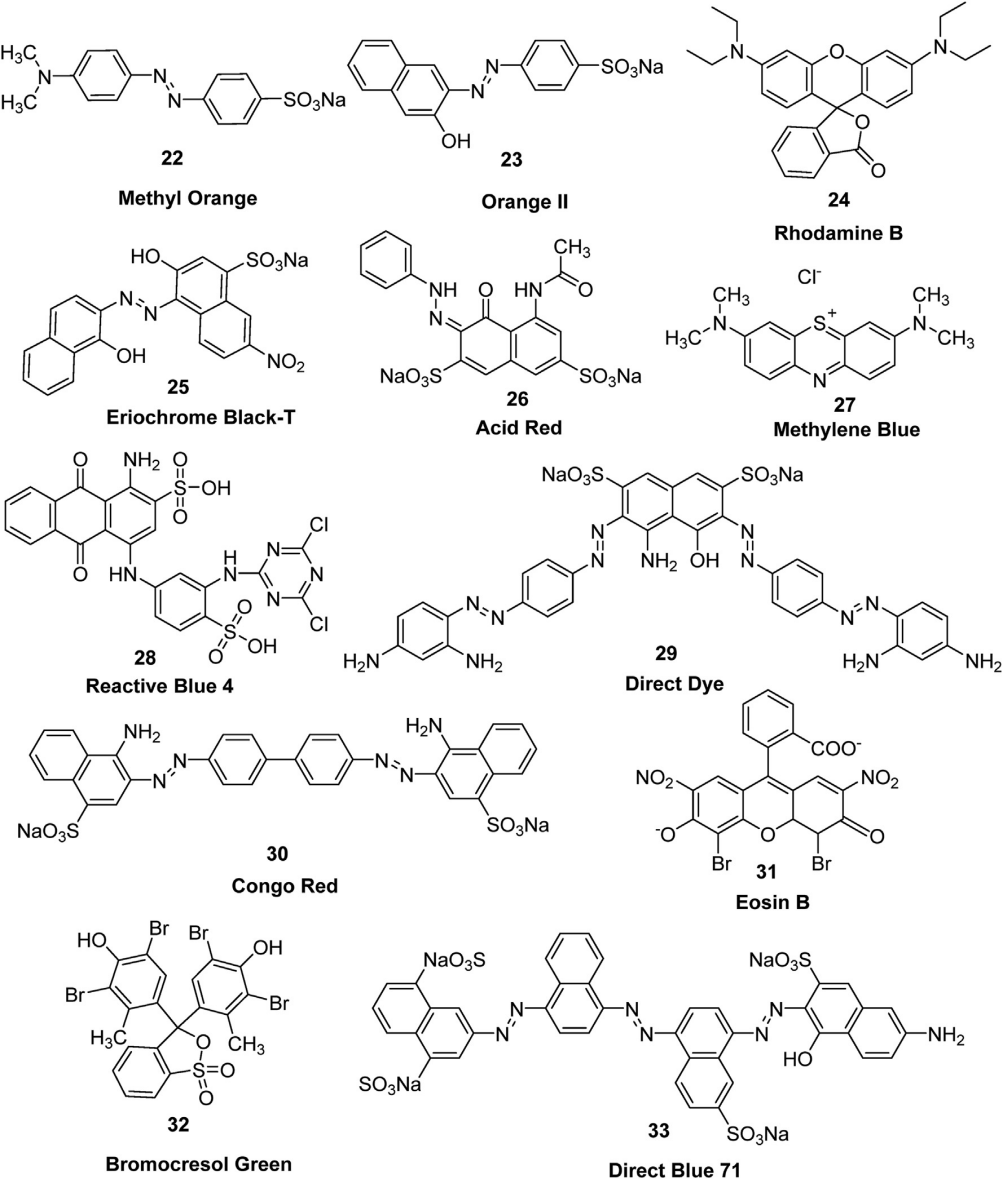
Dye molecules studied for the nZVC catalyzed removal process.

Dyes are another category of organic contaminants which pose a serious threat to sustainable health and the environment due to their non-biocompatible, carcinogenic, and chemically resistant nature. Dyes are excessively produced and used in various industries like tannery (Zhao and Chen, 2019), textiles (Mani and Bharagava, 2016), paints (F D Guerra et al., 2018) and cosmetics (Chaudhary, 2020), etc. These colored compounds not only impart color to the water bodies, but are a threat to aquatic life and life lying higher in the food web including humans (Tkaczyk et al., 2020). In this section, we will discuss the literature reports on the use of zerovalent copper for the removal of dyes (Figure 6) (Table 2).

**Table 2:** nZVC mediated removal of dyes molecules.

nZVC (bare or supported)	Pollutant	Oxidizing /Reducing agent (Conc.)	Optimum experimental conditions				Removal (%)	Ref.
			nZVC dose	Pollutant conc.	pH	Time		
Bare	Methyl Orange	-	40 mg^.^L^−1^	10 mg^.^L^−1^	3	20 min.	3%	(Li et al., 2015)
Bare	Congo Red Methyl Orange	NaBH_4_ (1.25 g.L^−1^)	1.4 g.L^−1^	0.03 mM	-	5 min.	90%	(Ismail et al., 2019)
Bare	Reactive Blue 4	-	1 g.L^−1^	15 mg^.^L^−1^	3	10 min.	90%	(Marcelo et al., 2018)
TiO_2_, chitosan, filter paper	Textile dyes	NaBH_4_ (0.5 g^.^L^−1^)	-	0.05 mM	3	5 min.	98.2%	(Alani et al., 2021)
Cellulose filter paper	Congo Red Methyl Orange	NaBH_4_ (0.26 g^.^L^−1^)	-	8.36 mg^.^L^−1^ 0.98 mg^.^L^−1^	-	13 min. 8 min.	100%	(Kamal et al., 2016)
Carboxymethyl cellulose	Methylene Blue 4-Nitrophenol	NaBH_4_ (0.26 g^.^L^−1^)	-	80 mg^.^L^−1^ 35 mg^.^L^−1^	-	10.6 h	100%	(Kamal et al., 2019)
Bare	Direct Black	-	1 g^.^L^−1^	20 mg^.^L^−1^	6.5	10 min	43%	(Ourique et al., 2018)
Bare	Eosin B	NaBH_4_ (0.37 g^.^L^−1^)	0.1 mg	58 mg^.^L^−1^	5	0.27 min.	100%	(Soomro et al., 2014)
Bare	Reactive Blue 19	-	80 mg^.^L^−1^	0.3 g^.^L^−1^	10	75 min	92.5%	(Haque et al., 2020)
Bare	Methyl orange Congo red Methylene blue Rhodamine B	-	0.3 g^.^L^−1^	20 mg^.^L^−1^ 20 mg^.^L^−1^ 10 mg^.^L^−1^ 5 mg.L^−1^	6.6 7.2 7	4 h 4 h 168 h 168 h	56.1% 59.6% 59% 95%	(Dong et al., 2014)
Fe(0)	Acid Orange 7	-	40 g^.^L^−1^	350 g^.^L^−1^	6.5	10 min.	94.3%	(Yuan et al., 2014)
Bare	Orange II	PMS (0.2 g^.^L^−1^)	0.3 g^.^L^−1^	8.75 mg^.^L^−1^	3	10 min.	96%	(Liu et al., 2021b)
Bare	Direct Blue 71	H_2_O_2_	1 g^.^L^−1^	50 mg^.^L^−1^	2.5	20 min.	78%	(Ertugay and Acar, 2013)
Carbon nanotube	Congo red	PMS (0.2 g^.^L^−1^)	12.7 mg^.^L^−1^	10 mg^.^L^−1^	6.5	40 min.	100%	(Zheng et al., 2021)

#### 5.2.1 Azo dyes as pollutants

The azo dyes constitute nearly 70% among the commercial dyes used in textile and dye industries around the world (Phugare et al., 2011). Further, it has been estimated that nearly 50% of the total dye content employed for the dyeing process do not bind with the fabric and are released as effluent in industrial wastewater (Rehman et al., 2018). Azo dyes are highly toxic and carcinogenic in nature and prove harmful for aquatic life by increasing the biochemical oxygen demand (BOD) of water (Liu et al., 2017; Phugare et al., 2011). Therefore, there is an utmost need for remediation of these colored harmful contaminants.

Zerovalent copper nanoparticles in the Fenton like process were used for the degradation of methyl orange (MO) azo dye **22** (Li et al., 2015). Hydrodynamic cavitation significantly increased the rate of decolorization of methyl orange by preventing ZVC agglomeration and promoting the generation of hydroxyl radicals in the reaction mixture. Surface adsorption of azo dye onto ZVC was followed by hydrogenation under acidic conditions (pH 3) giving aromatic amines. At the same time, hydroxyl radicals generated from extremely high temperatures resulting from adiabatic compression of cavities causes degradation of dyes. The MO (10 mg^.^L^−1^) degradation efficiency of 83% was achieved under optimum reaction conditions including ZVC dose of 40 mg^.^L^−1^, pH 3.0, and 0.4 MPa discharge pressure within 20 min of reaction time. The pseudo first order rate kinetics was observed for the MO degradation, which increased linearly with the ZVC dose. The reaction intermediates were confirmed from Fourier transform IR (FT-IR) analyses and UV-Vis spectroscopy.

Cu/Fe bimetallic system was prepared by depositing Cu^0^ on Fe^0^ surface by a simple metal displacement reaction observed due to a large standard reduction potential difference between Copper and iron i.e. E_o_^r^ is 0.78 V (Yuan et al., 2014). Fe/Cu bimetallic particles exhibits a significant Acid Orange 7 (AO7) **23** removal efficiencies (94.3%) under optimal reaction condition of Cu@Fe conc. of 40 g·L^−1^, AO7 conc. of 1000 mM at initial solution pH of 6.5 within 10 min of reaction time. UV-Vis analysis and FTIR studies confirmed the destruction of chromophore part (-N=N-) that results in formation of 1-amino-2-naphthol and sulfanilamide as degradation intermediates, which are further mineralized during the process or are precipitated with Fe ions. In another study performed by Liu et al. (2021b), the ZVC coupled Fe^2+^ ions promoting PMS activation process was used to carry out the degradation of orange II **23** dye. The Cu(III), ^•^OH and SO_4_^•-^ was the species identified for the orange II oxidative degradation. Under acidic conditions, ZVC produces Cu^+^ which reacts with molecular oxygen and produces H_2_O_2_. Then both Cu^+^ and Fe^2+^ induced a Fenton like process to decompose H_2_O_2_ and PMS to give ^**.**^OH and SO_4_^•-^ radicals to cause degradation of orange II dye. Other than orange II dye, the ZVC/Fe(II)/PMS system was able to achieve a degradation efficiency of more than 90% for rhodamine B **24**, methyl orange **22**, acetaminophen **41**, propranolol **49**, ibuprofen **45** and diclofenac **42**.

Ismail et al. (2019) synthesized the stabilized zerovalent copper nanoparticles (CuNPs) using a green process employing the fruit extract of the *Duranta erecta* plant, as evidenced by the emergence of an absorption band at 588 nm in the UV-Vis spectrum. The CuNPs were tested for their efficiency in the reductive degradation of carcinogenic azo dyes such as congo red (CR) **30** and methyl orange **22** using NaBH_4_, with rate constant values of 5.0710 s^−1^ and 8.610 s^−1^, respectively, based on pseudo-first-order kinetics. In another study, Liu et al. (2016) used visible light to perform zerovalent copper catalyzed photo-degradation of methyl orange dye in aqueous solution. The results obtained from simulation using finite-difference time-domain (FDTD) method suggests that the surface electric charge increases with absorption of light and with decrease in size of nZVC. This results in enhanced photo-catalytic efficiency observed for nZVC for MO degradation.

Zheng et al. (2021) synthesized ZVC supported carbon nanotubes (nZVC–CNT) via simple chemical reduction of pre-adsorbed Cu^2+^ to Cu^0^ using NaBH_4_ as a reducing agent. The nZVC–CNT nanocomposite was effectively used as a Fenton-like advanced oxidation process (AOP) via activation of peroxymonosulfate (PMS) to carry degradation of congo red dye **30**. The AOPs system having electrocatalytic membrane was found more effective compared to the conventional batch system. The CNT’s carbonyl group (C=O) served as electron donor, while electrophilic oxygen served as electron acceptor to activate PMS to generate hydroxyl radicals (^**.**^OH) and singlet oxygen (^1^O_2_) to initiate the congo red degradation.

Metallic copper nanoparticles (CuNPs) prepared via reduction route were compared with zinc nanoparticles (ZnNPs) for their degradation efficiency of aqueous Acid Red dye (AR dye) **26**. The CuNPs at its lower dose (30 mg) were found effective in the degradation of AR dye compared to ZnNPs (80 mg). The experimental data was best fit with a pseudo-second-order kinetic model with higher correlation coefficients. The removal process involves reductive catalytic degradation of AR dye with both CuNPs and ZnNPs. The catalysts were found effective in treatment of real wastewater samples containing AR dye (Salam et al., 2019).

Kamal et al. (2016) loaded zerovalent copper nanoparticles on microfibrous cellulose filter paper coated with chitosan (1%) to give Cu/CH-FP nanocomposite material and investigated for degradation studies of congo red **30** and methyl orange **22** dyes using NaBH_4_ as reducing agent. The significant rate constant values of 0.1655 and 0.2683 min^−1^ for the CR and MO suggested the good catalytic activity of the composite, which was further complemented with easy removal of dip-like Cu/CH-FP strip from the reaction medium and its reusability with high removal efficiency (75%) even after several cycles. In another studies, carboxymethyl cellulose stabilized nZVCs immobilized on bacterial cellulose nanofiber were synthesized via microwave synthesis and were investigated for their catalytic reduction efficiency of mixed solution of methylene blue **27** and 4-nitrophenol **12** using NaBH_4_ as reducing agent (Kamal et al., 2019). Although the catalytic performance of bacterial cellulose nanofiber supported nZVC was lower than the carboxymethyl cellulose stabilized nZVC suspension, however, the ease of removal of the supported nZVC make it more convenient to use as heterogeneous catalyst.

Ourique et al. (2018) investigated the degradation efficiencies of ZVC, nZVI and Cu–Fe bimetallic systems towards direct dye from aqueous solutions. The FeNP (100%) and Fe–Cu bimetallic system (90%) were found more effective for the direct dye **29** removal under anoxic conditions compared to ZVC (40%) suggesting the reductive degradation mechanism under conditions studied.

Dong et al. (2014) studied the role of zero-valent copper in activating O_2_ via electron transfer process at neutral pH to generate different reactive oxygen species (ROS) and release of Cu^+^ species to participate in Sandmeyer type reaction to break –N=N- bond of azo dyes like methyl orange **22** and congo red **30** to generate carbon centered radicals. The different organic dyes studied were methyl orange **22**, congo red **30**, methylene blue **27** and rhodamine B **24**. The ^**.**^OH radicals generated from H_2_O_2_ destruction then reacted with carbon centered radicals causing transformation of organic compounds to low molecular weight acids with subsequent mineralization. GCMS technique confirmed the presence of different degradation fragments resulting from carbon centered radical destruction processes of different azo dyes. ESR confirmed the presence of ^**.**^OH and O_2_^**.-**^ radical species in the reaction conditions.

Ertugay et al. (2013) performed the ZVC mediated sonocatalytic degradation of Direct Blue 71 (DB71) **33** under acidic conditions at 20 kHz with ultrasound power of 95W. Approximately 55.8% dye (50 mg^.^L^−1^) removal was achieved under optimum reaction conditions of 1 g^.^L^−1^ of catalyst at initial pH of 2.5 at 20 °C within the reaction time of 20 min. The dye removal efficiency was further increased to 78% in the presence of H_2_O_2_ as an oxidizing agent under reaction conditions.

#### 5.2.2 Anthraquinone dyes as pollutant

After azo dyes, the anthraquinone based dye molecules are the second largest organic dye compounds produced worldwide for coloring of textile fabrics. Owing to their complex and reinforced structure, the anthraquinone based dyes provide a natural resistance to degradation process and thus pose a serious threat to the environment (Routoula et al., 2020).

Zero-valent copper nanoparticles (nZVC) were also investigated for the removal of the Reactive Blue 4 dye (Marcelo et al., 2018). Approx. 90% of the oxidative degradation with concomitant mineralization of RB4 dye was achieved within the 10 min of reaction time as confirmed from Total Organic Carbon (TOC) analysis. Cu(I) mediated oxidative process was responsible for the removal process instead of ^·^OH radicals as confirmed from the use of tert-Butyl alcohol as ^·^OH captor. The reaction rate was decreased with increase in initial concentration of dye and increased with decrease in pH (from 7 to 3), increase in temperature (from 10 to 30 °C) and increase in nZVC dose (from 0.5 to 2 g^.^L^−1^). Experimental data was best fit using a second-order kinetics model and activation energy determined was 42 kJ mol^−1^. The nZVC nanoparticles exhibit good recyclability without any significant loss of RB4 removal efficiencies. A greener approach involving the use of fish scales of *Labeo rohita* was employed for the synthesis of zerovalent copper and later was applied for the decolorization reaction of Reactive Blue 19 dye **28** (Haque et al., 2020).

#### 5.2.3 Fluorescein dyes as pollutant

Fluorescein dyes are used as fluorescent tracers and have many applications in biological systems. These are considered non-toxic, non-pollutant and non-carcinogenic, however, there release in water system increases the biochemical oxygen demands (BOD). Alani et al. (2021) synthesized the zero-valent copper nanoparticles immobilized onto a porous support consisting of TiO_2_, Chitosan and filter paper to produce nanocomposite material (Cu/CHTiO_2_/FP). The nanocomposite has pronounced catalytic efficiency for textile dyes like rhodamine B **24**, methyl orange **22**, eriochrome black-T **25** and bromocresol green **32** using NaBH_4_ as a reducing agent. It’s easy recovery and reusability with high dye removal efficiency (> 90%) even after five cycles make it an efficient material for remediation of colored compounds.

Soomro et al. (2014) studied the sodium dodecyl sulfate (SDS) capped ZVC for reductive degradation of Eosin B (EB) dye **31**. Approximately 100 % removal efficiency of EB (100 μM) was achieved within a reaction time of 20 s using 500 μL of NaBH_4_ (10 mM) and 0.1 mg ZVC catalyst. The enhanced catalytic activity of nanocatalyst was a result of rough surface evident from AFM analysis, providing a greater number of active sites for reaction.

In another study performed by Ghanbari et al. (2014) the sulfate radicals generated by zerovalent iron and copper mediated activation of peroxymonosulfate was used for the decoloration of textile wastewater. ZVI was found to be more effective for TOC and COD removal when compared to ZVC. However, among the ZVC mediated activation of PMS and H_2_O_2_ for the decoloration of textile wastewater, the former was proved more significant. Further, the simultaneous employment of PMS and H_2_O_2_, synergically enhanced the decoloration efficiencies of both ZVC and ZVI.

### 5.3 Removal of drugs

**Figure 7:**
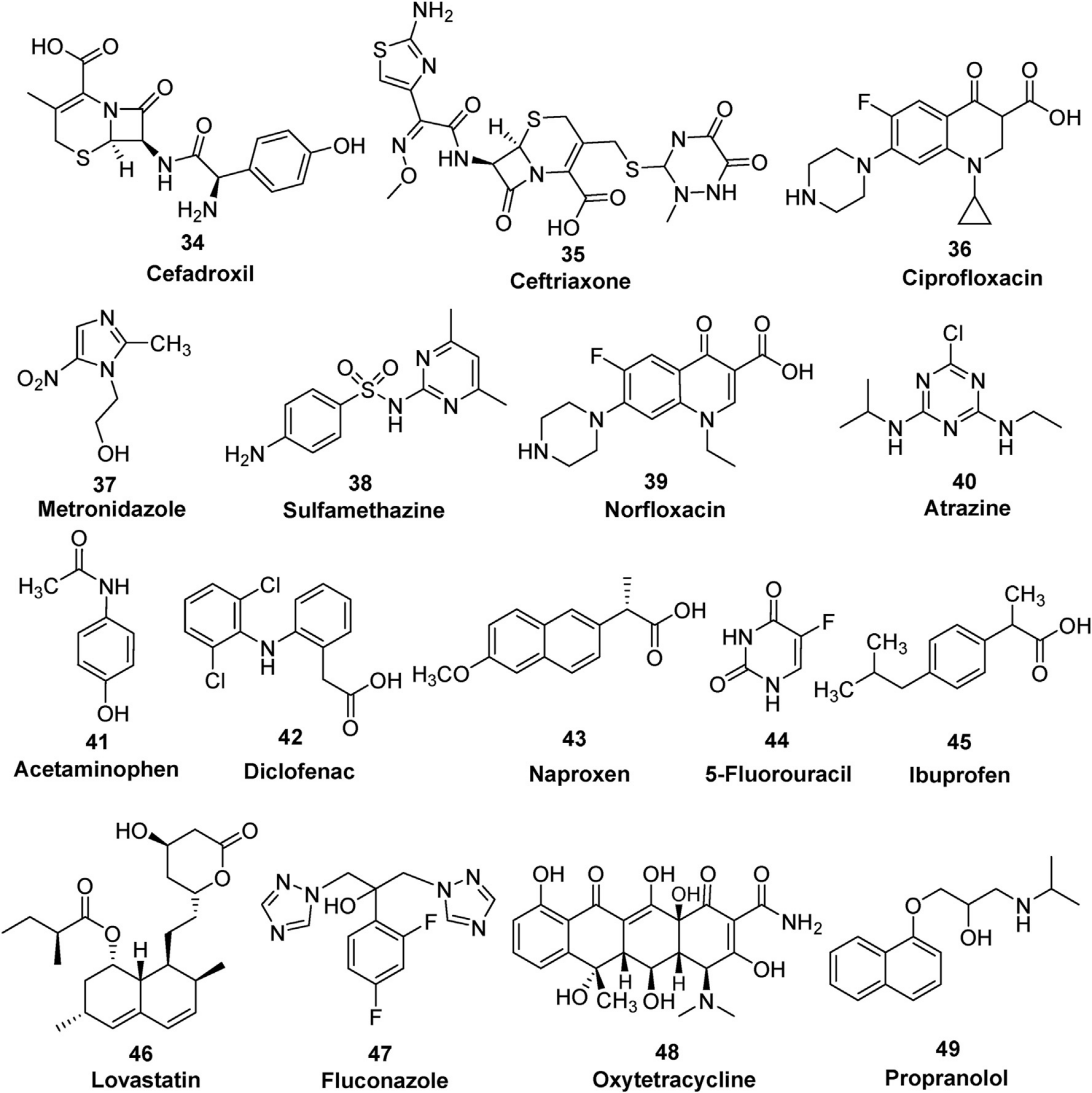
Pharmaceuticals studied for the nZVC catalyzed removal process.

Pharmaceutical products or drugs are another category of organic contaminants, which enter the ecosystem through different pathways extending from pharmaceutical industrial disposal to metabolites of human healthcare medicines. Due to their persistent nature, they may prove harmful to aquatic life and also to humans. This part of the discussion will present literature reports employing zerovalent copper used for the removal of pharmaceutical products (Figure 7) (Table 3).

**Table 3:** nZVC mediated removal of drug molecules.

nZVC (bare or supported)	Pollutant	Oxidizing /Reducing agent (Conc.)	Optimum experimental conditions				Removal (%)	Ref.
			nZVC dose	Pollutant conc.	pH	Time		
Bare	Cefadroxil Ceftriaxone	-	1 g^.^L^−1^	76 mg^.^L^−1^ 50 mg^.^L^−1^	9.5	20 min.	85% 97%	(Oliveira et al., 2018)
Bare	Ciprofloxacin	-	0.5 g^.^L^−1^	20 mg^.^L^−1^	3.5	2h	100%	(de Sousa et al., 2019)
Graphene	Metronidazole	-	0.5 g^.^L^−1^	20 mg^.^L^−1^	3.2	20 min.	92%	(Xu et al., 2019)
Bare	Acetaminophen	-	5 g^.^L^−1^	50 mg^.^L^−1^	3	4h	100%	(Zhang et al., 2017b)
Bare	Norfloxacin	H_2_O_2_ (0.68 g^.^L^−1^)	0.25 g^.^L^−1^	5 mg^.^L^−1^	-	30 min.	92%	(Ma et al., 2018)
Bare	Norfloxacin	PS (0.22 g^.^L^−1^)	0.05 g^.^L^−1^	10 mg^.^L^−1^	-	5 min.	100%	(Deng et al., 2019)
Bare	Sulfamethazine	PS (0.11 g^.^L^−1^)	64 mg^.^L^−1^	5 mg^.^L^−1^	3.06	60 min.	100%	(Zhang et al., 2020b)
Montmorillonite	Atrazine	-	0.5 g^.^L^−1^	12.5 mg^.^L^−1^	3.0	2 min.	90%	(Hong et al., 2017)
Bare	Acetaminophen	PMS (0.2 g^.^L^−1^)	0.5 g^.^L^−1^	7.5 mg^.^L^−1^	3.0	12 min.	89%	(Liu et al., 2021a)
Bare	Diclofenac	PAA (7.6 g^.^L^−1^)	0.5 g^.^L^−1^	0.30 mg^.^L^−1^	3.0	40 min.	95.5%	(Zhang et al., 2021)
Graphene	Naproxen	PMS (0.3g^.^L^−1^)	2 g^.^L^−1^	1.15 mg^.^L^−1^	3.0	30 min.	91%	(Chi et al., 2019)
Bare	5-Fluorouracil Lovastatin	-	25 mg^.^L^−1^	50 mg^.^L^−1^	6.8	90 min.	65.1% 78.19%	(Dinesh et al., 2020)
Vanadium-doped	Fluconazole	H_2_O_2_ (1.7 g^.^L^−1^)	1 g^.^L^−1^	20 mg^.^L^−1^	3	60 min.	100%	(Zhang et al., 2020a)
Fe_3_O_4_	Oxytetracycline	H_2_O_2_ (0.6 g^.^L^−1^)	1 g^.^L^−1^	20 mg^.^L^−1^	3	10 min.	97%	(Kim and Ko, 2018)

Oliveira et al. (2018) studied the nZVC for the removal of cefadroxil **34** and ceftriaxone **35** antibiotics from aqueous solution under oxic and anoxic conditions. More than 85% of the antibiotics were removed within a reaction time of 20 min. Use of tert-butyl alcohol as a radical inhibitor confirmed that the hydroxyl radicals is not the sole requirement of the antibiotic degradations, but the presence of Cu^+^ was responsible for the overall removal process. Two-step removal process was observed, whereby the Cu^+^ species promoted degradation of antibiotics was deliberated as the first step followed by adsorption of antibodies on the copper oxides/hydroxides in the second step. Both these removal steps followed pseudo first order kinetic models. Nearly 57% of cefadroxil removal was obtained within 180 min as observed from TOC analysis.

In another study, degradation of ciprofloxacin **36** by metallic copper nanoparticles was investigated under aqueous conditions (de Sousa et al., 2019b). The optimum conditions involve 20 mg^.^L^−1^ ciprofloxacin concentrations, 0.5 g^.^L^−1^ nZVC dose with solution pH of 3.5. Under acidic conditions, Cu(I) mediated formation of active oxygen radicals were responsible for the degradation of ciprofloxacin. However, adsorption and coprecipitation were observed as primary phenomena for the removal of antibiotics under the basic conditions, which was further confirmed from the desorption experiments. Increase in dose concentration, temperature and chloride anions favors the degradation kinetics, whereas increase in sulfate anion concentration has inhibitory effect on the ciprofloxacin degradations. A reduction in reusable efficiency (70% removal) was observed due to surface passivation of nZVC nanoparticles due to formation of Cu_2_O in consecutive cycles.

A self-assembly involving the liquid-phase reduction process was used for the synthesis of 3D-macroporous graphene-wrapped nZVC nanocomposite (3D-GN@Cu^0^). The composite material displayed high efficiencies for the degradation of aqueous metronidazole **37** saturated with dissolved oxygen under various pH conditions ranging from 3.2 to 9.8, without using H_2_O_2_ as oxidizing agent (Xu et al., 2019). Activation of dissolved oxygen to surface bounded ^·^OH via Fenton-like process was observed from the XPS analysis of the sample and held responsible for the degradative removal of metronidazole. The DFT calculation was used to justify the results obtained from micro-electrolysis of 3D-GN@Cu^0^ and also explained the observed synergistic effect between graphene and nZVCs for the removal of metronidazole.

Zhang et al. (2020b) performed sulfamethazine (SMZ) **38** degradations using a synergistic approach involving nZVC and sonolysis promoted activation of persulfate to give SO_4_^•-^. The optimal conditions for the complete SMZ removal includes a PS dose of 0.5 mM, nZVC conc. of 64 mg^.^L^−1^, reaction time of 60 min, ultrasound energy of 0.4 W/mL and 40 kHz at solution pH of 3.06. The inhibitory effect was observed with anions like sulfate, nitrate, bicarbonate and chloride ions. The major degradation pathways proposed includes S–N bond cleavage, SO_2_ extrusion (Smile rearrangement), and oxidative degradation of aniline moiety.

**Figure 8:**
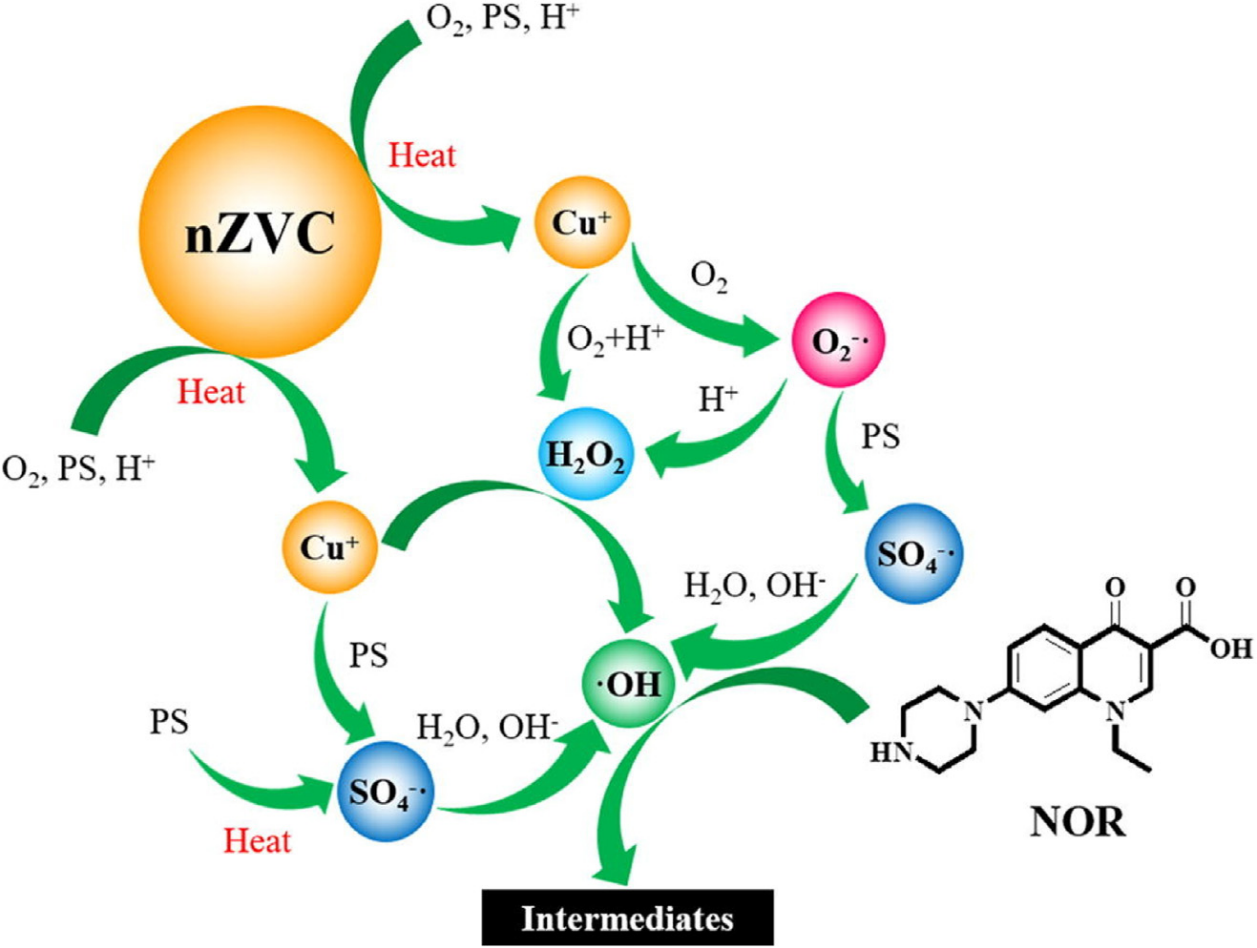
nZVC mediated activation of persulfate and degradation of norfloxacin (Deng et al., 2019).

Degradation analysis of norfloxacin (NOR) **39**, a fluoroquinolone based antibiotic was performed by Deng et al. (2019) using nZVC/PS system with application of mild temperature (40 °C). The temperature enhanced the release of Cu^+^ from nZVC in the solution promoting the formation of ^**.**^OH and SO_4_^-.^ species responsible for degradation of norfloxacin. In another study, Ma et al. (2018) used a synergistic effect between ZVC and ultrasonic irradiation (US) to activate H_2_O_2_ to induce a Fenton-like reaction. Nearly 100% degradation was observed within 30 min. of the reaction time using 0.25 g^.^L^−1^ of nZVC, 10 mM conc. of H_2_O_2_ with US of 240 W at 20 kHz. EPR analysis confirmed the presence of ^**.**^OH radicals as the primary species responsible for NOR degradation and superoxide radicals (O_2_^**.-**^) as a mediator for regeneration of Cu^+^ from oxidized form of copper i.e. Cu^2+^. The oxidative cleavage of piperazine moiety and transformation of quinolone was regarded as a major degradation step among other reported oxidation pathways (Figure 8).

Hong et al. (2017) evaluated the montmorillonite clay supported zerovalent copper (ZVCMMT) for the degradation analysis of atrazine **40**. The montmorillonite supported ZVC exhibited a significant atrazine removal efficiency (90%) compared to conventionally prepared ZVCs. Further the hydration status of prepared nanocomposite strongly determines the atrazine degradation efficiency by controlling the adsorption of oxygen and atrazine residues on the freeze dried surface of the nanocomposite. The prepared ZVCMMT nanocomposite significantly decreased the toxicity of atrazine in the studied reaction conditions.

**Figure 9:**
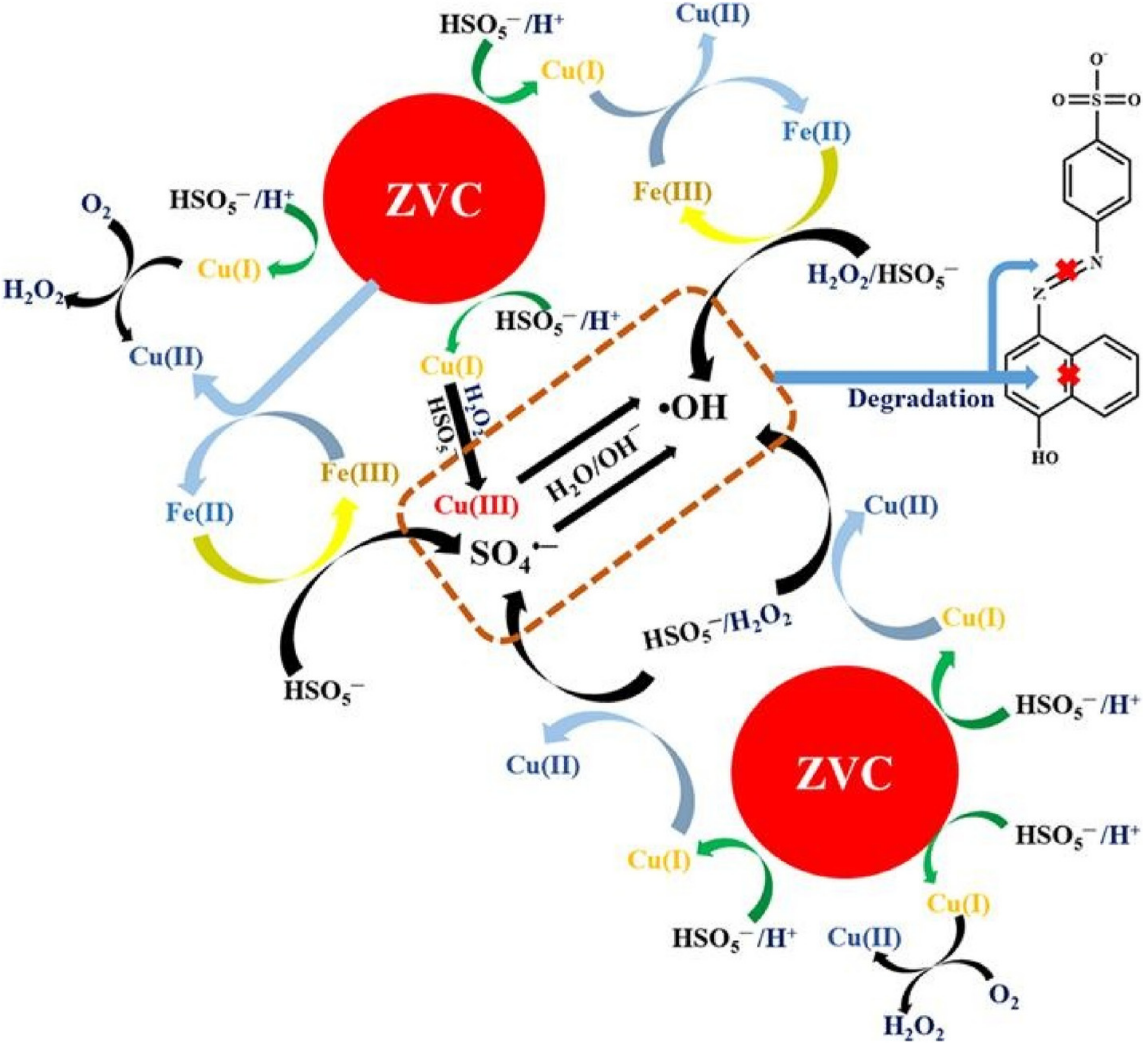
Schematic representation of acetaminophen removal by Fe(III)/Cu(0)/PMS system (Liu et al., 2021a).

Zhang et al. (2017b) used commercially synthesized ZVC for the investigation of acetaminophen (ACT) **41** degradation by *in-situ* generated reactive oxygen species (ROS) like H_2_O_2_, hydroxyl radical (^**.**^OH) and superoxide anion radical (O_2_^**.-**^) under acidic conditions (pH 3). The Cu^+^ ion generated from ZVC under acidic conditions not only produces H_2_O_2_ via activation of O_2_, but also helped in its decomposition to produce ^**.**^OH species, actually involved in the degradation process of acetaminophen and further confirmed by ESR analysis. The superoxide anion radical (O_2_^**.-**^) helped in the regeneration of Cu^+^ from its oxidized forms i.e. Cu^2+^ via one electron transfer process. In another study performed by Liu et al. (2021a) the Fe^3+^ addition to the ZVC/PMS significantly enhanced the acetaminophen degradation. ZVC promotes PMS oxidation under acidic conditions by single electron transfer resulting in formation of Cu^+^. The Fe^3+^ interacts with both Cu^+^ and Cu^0^ to generate Fe^2+^, which is considered more active compared to Cu^+^ in generation of ^**.**^OH radicals from the Fenton-like process. In the final step, both Cu^+^ and Fe^2+^ activate H_2_O_2_ and PMS for the acetaminophen degradation (Figure 9). Common ions in water including sulfate, carbonate, and nitrate had no adverse effect on acetaminophen degradation process by Fe(III)/ZVC/PMS system, whereas humic acid and chloride ions slightly inhibit the acetaminophen degradation process.

Zhang et al. (2021) used zerovalent copper for the activation of peracetic acid to give radical species like ^.^OH, CH_3_COO^.^ and CH_3_COOO^.^ to study the degradation profile of sodium salt of diclofenac **42**. Corrosion of ZVC to Cu^+^ species at pH 3 was responsible for the activation of peracetic acid, hydrogen peroxide and molecular oxygen. UPLC-QTOF/MS analysis suggested seven different degradation pathways depending on the detection of six different degradation products as: (i) hydroxylation; (ii) amidation; (iii) dechlorination-cyclizaiton; (iv) dechlorination-hydrogenation; (v) dechlorination-hydroxylation; (vi) decarboxylation; and (vii) formylation.

**Figure 10:**
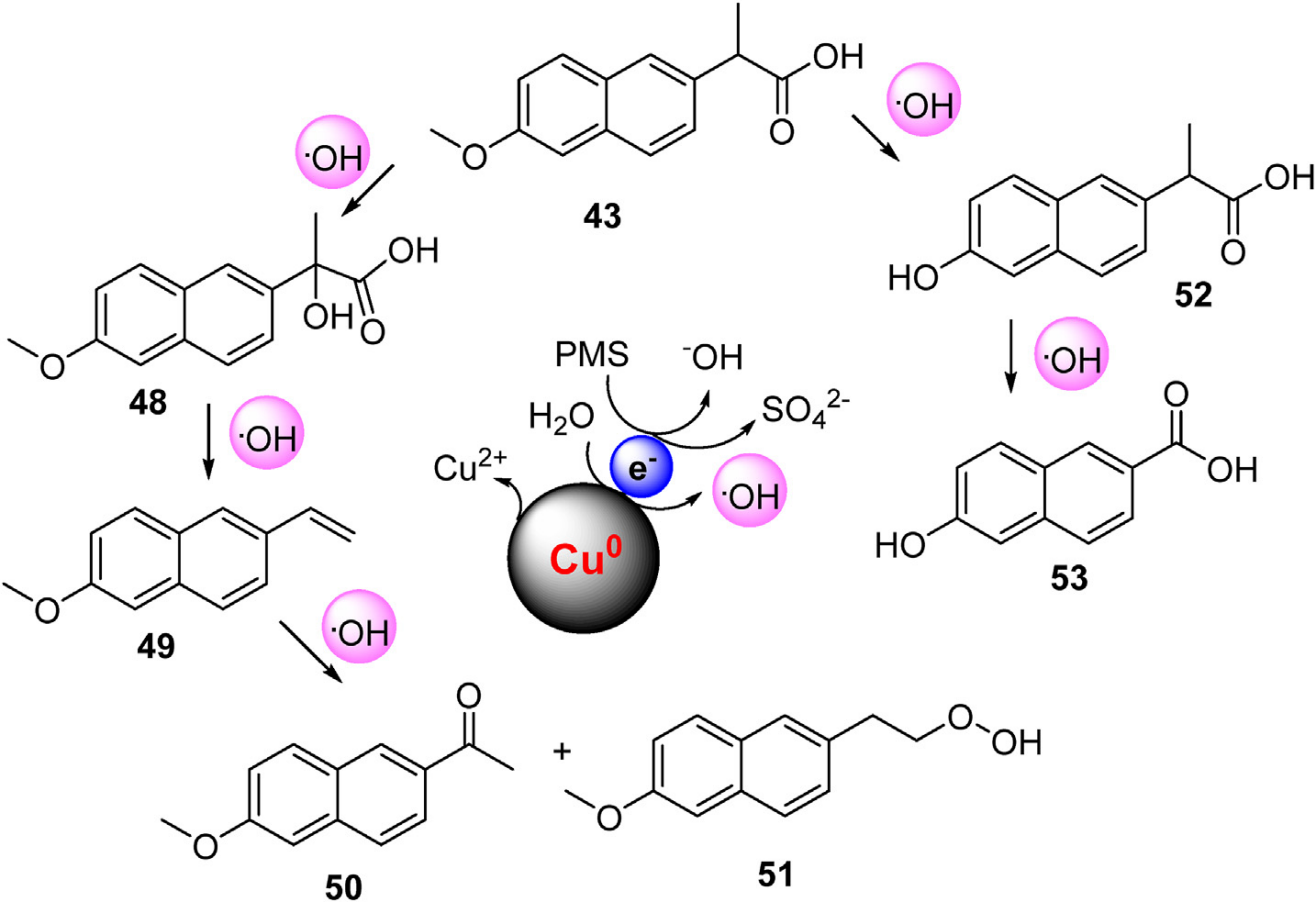
Proposed pathways for naproxen **43** degradation in ZVC-PMS activation system (redrawn from Chi et al., 2019).

Chi et al. (2019) employed the ZVC-activated PMS system for the evaluation of the degradation mechanism of naproxen **43**, an anti-inflammatory drug. Four different forms of ZVC have been employed for the PMS activation namely copper sheet, copper foam, graphene-copper sheet, and graphene-copper foam. ZVC coating with graphene results in an increase in naproxen degradation efficiency by 10% and decrease in Cu^2+^ release by 30%. ESR analysis and radical scavenging studies confirmed that hydroxyl radicals were the dominant species responsible for degradation other than sulfate radicals. On the basis of HPLC-MS/MS analysis, six different intermediates were identified and thus helped in proposing the naproxen degradation pathway (Figure 10).

**Figure 11:**
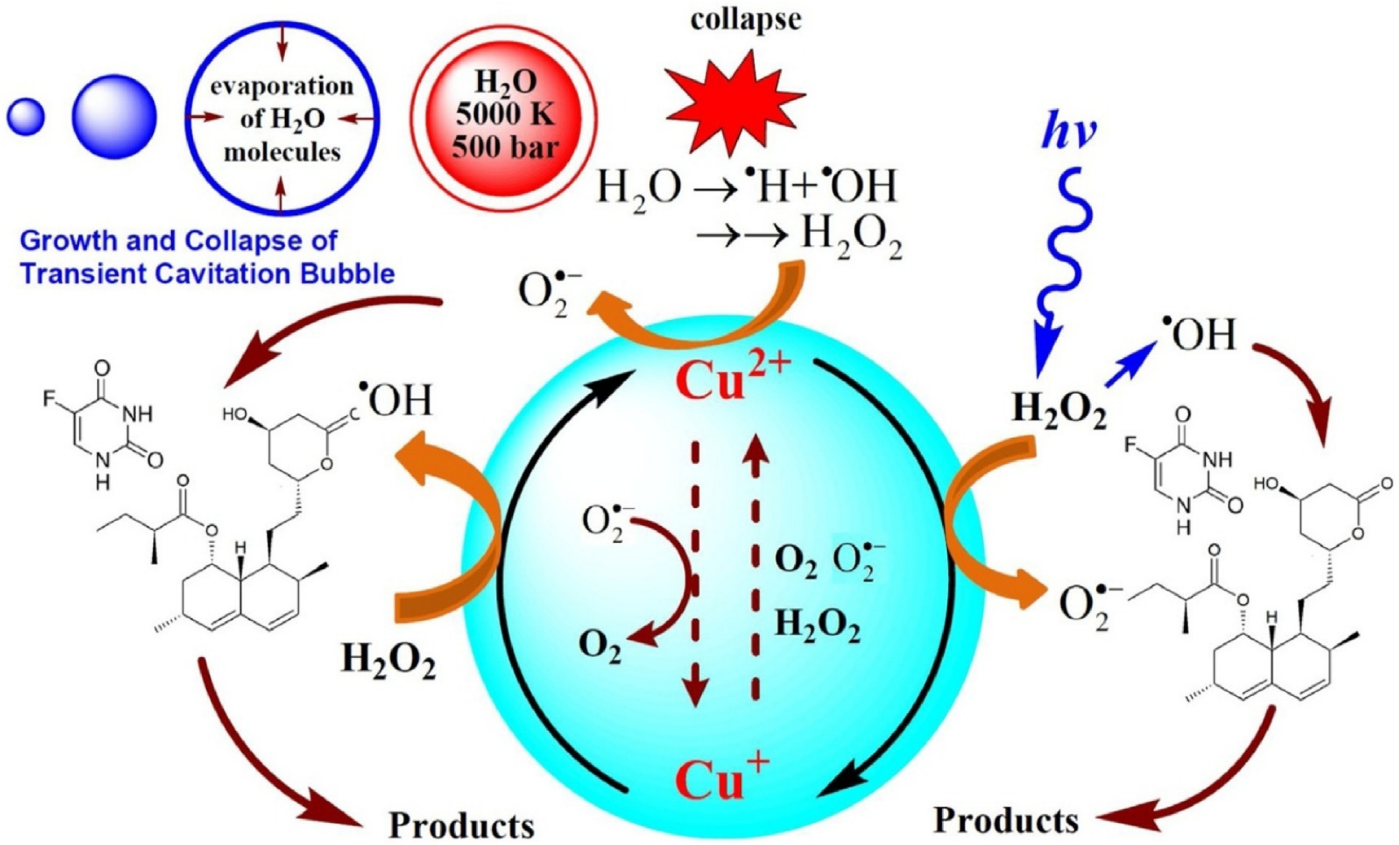
Ultrasound assisted degradation mechanism of Lovastatin **46** and 5-Fluorouracil **44** drug molecules in the presence of nZVC (Dinesh et al., 2020).

The *Hibiscus Rosa-Sinensis* extract as a reducing as well as stabilizing agent for the synthesis of ZVC nanoparticles (Dinesh et al., 2020). The ultrasound energy helped in controlling the crystallinity and size of the ZVC nanoparticles during the synthesis process. The synthesized ZVC displayed amphoteric nature with dual catalyst activation mechanism in the presence of UVC-light or ultrasound irradiation. The ZVC nanoparticles displayed high degradation efficiencies of 91.3% and 93.2% for 5-fluorouracil **44** and lovastatin **46** drugs, respectively. The hydroxyl radicals generated from UV irradiation combined with the sonolysis process and oxidation of Cu^+^ to Cu^2+^ during ultrasound irradiation were responsible for degradation of drugs. The degradation process was further enhanced by superoxide radical (O_2_^**.-**^) produced from reaction of in-situ generated H_2_O_2_ with Cu^2+^ (Figure 11).

Zhang et al. (2020a) investigated the removal efficiencies of Cu–V bimetallic catalyst, prepared via hydrothermal approach, towards fluconazole **47** an active antifungal pharmaceutical product. Addition of vanadium to copper material not only improved its surface properties like number electron-rich center around active sites, surface defects and adsorption capacities that result into enhancement of catalytic efficiencies of bimetallic system, but also helped in decreasing Cu^2+^ concentrations by reducing it back to give active Cu^+^ species. The surface oxygen vacancies helped in easy destruction of *in-situ* generated H_2_O_2_ via Fenton-system to give ^.^OH radicals responsible for the oxidative degradation of fluconazole. The EPR analysis and radical scavenging test further confirmed the involvement of ^**.**^OH radicals.

*A core*-shell structure based Cu@Fe_3_O_4_ nanocomposite consisting of ZVC as core and Fe_3_O_4_ as protective shell was synthesized by Kim et al. (2018) using a simple reduction method. Cu@Fe_3_O_4_ displayed exceptional synergistic effect in oxidative degradation of oxytetracycline **48**, an antibiotic used in personal care products via a Fenton-like reaction. More than 99% oxytetracycline (20 mg^.^L^−1^) degradation was achieved within 10 min of the reaction time using 1 g^.^L^−1^ dose of Cu@Fe_3_O_4_, 20mM conc. of H_2_O_2_ at pH 3 and pH 9.

### 5.4 Removal of heavy metals

Heavy metals are considered highly toxic due to their high degree of bioaccumulation efficiencies (Bonsignore et al., 2018; Vajargah, 2021) and the role it plays in the inhibition of various enzymatic activities in humans that result in organ damage (Fu and Xi, 2020; Lopina, 2017). Although the reports are limited, zerovalent copper has been effectively used for the remediation of heavy metals from aqueous medium (Table 4).

**Table 4:** nZVC mediated removal of heavy metals.

nZVC (bare or supported)	Pollutant	Optimum experimental conditions				Removal (%)	Ref.
		nZVC dose	Pollutant conc.	pH	Time		
Chitosan	Chromium	1 g^.^L^−1^	50 mg^.^L^−1^	2.85	5 h	47.8	(Wu et al., 2009)
Bare	Uranium	0.03 g^.^L^−1^	0.1 mg^.^L^−1^	4	1 h	130	(Chandra and Khan, 2020)
Biochar	Lead	2.5 g^.^L^−1^	70 mg^.^L^−1^	7	12 h	29.57	(Din et al., 2021)
Pistachio shell powder	Chromium	0.1 g^.^L^−1^	20 mg^.^L^−1^	3	17 h	93.5	(Kumar et al., 2021)

Chitosan-tripolyphosphate containing chelating resin beads was used as a support for the synthesis of copper-chitosan composite material, in which the copper (II) ions were first adsorbed on to the surface of chitosan-tripolyphosphate beads followed by reduction using chemical process that results into well dispersed copper nanoparticles on the surface of chitosan beads (Wu et al., 2009). The prepared copper-chitosan composite material was compared with the chitosan-tripolyphosphate beads for their removal efficiencies of aqueous hexavalent chromium via adsorption, co-precipitation or redox mechanisms, and was found to have better adsorption efficiencies than later. The optimum conditions to obtain maximum adsorption of Cr(VI) involves copper-chitosan nanocomposite dose of 1 g^.^L^−1^ and Cr(VI) initial concentration of 50 mg^.^L^−1^ at pH 2.85. From EDS analysis it was evident that the adsorption of Cr(VI) was observed both on the surface (10.53 wt.%) as well as the inside (5.13 wt.%) of the copper-chitosan nanocomposite.

Uranium concentration in water if exceeds its permissible limits proves carcinogenic for humans and other living beings. *Nano zerovalent* copper synthesized by environment-friendly green method using testa extract of *Anacardium occidentale* was evaluated for the removal of uranium from aqueous solution (Chandra and Khan, 2020). Polyphenolic functional groups present in the *Anacardium occidentale* testa extract not only reduce the Cu(II) to Cu(0), but also act as stabilizing agent for the synthesized nZVCs. The maximum adsorption efficiency (96.63%) was achieved with nZVC dose of 0.03 g^.^L^−1^ for 100 ppb initial concentration of uranium with a reaction time of 60 min. at pH 4. The best fit of experimental data was obtained using Langmuir adsorption isotherm (*R*_*L*_ = 0.1733) and Freundlich adsorption isotherm (*R*^*2*^ = 0.99) models.

Din et al. (2021) used cotton stalk biochar as a support to synthesize zerovalent copper nanocomposite materials and tested for its adsorption abilities to remove lead from the aqueous solutions. Kinetic equilibrium studies suggest the pseudo-second-order to be followed with rate constant value of 720 min. for lead removal by ZVC composite. Study of thermodynamic parameters points towards the spontaneous but endothermic chemisorptive nature of the adsorption process.

**Figure 12:**
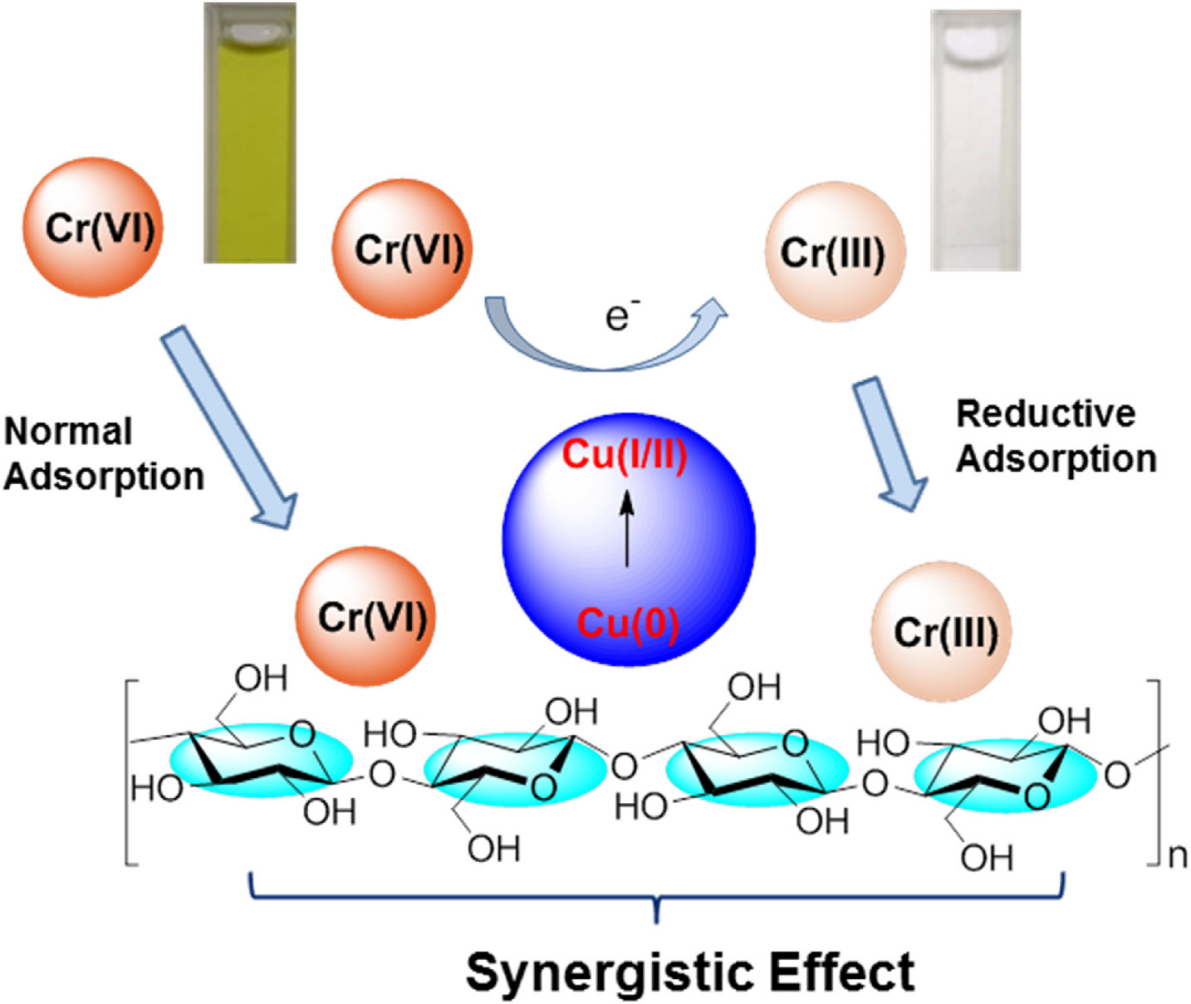
Schematic illustration of Cr(VI) removal by nZVC@PS using synergistic effect (Kumar et al., 2021).

Kumar et al. (2021) prepared the pistachio shell supported zerovalent copper (nZVC @PS) and studied its synergistic effect for the removal of Cr(VI) from the aqueous solutions using the adsorption efficiency of pistachio shell powder and reduction capacities of zerovalent copper (Figure 12). The nanocomposite exhibits significant Cr(VI) removal efficiencies (110.9 mg^.^g^−1^), when compared to other nanocomposite reports.

Overall, it may be concluded that the best removal efficiencies for nZVC towards various environment pollutants can be achieved under acidic pH 3–6, regardless of the target contaminant. However, the progressive explanations may vary in different contaminant removal studies, but the enhanced dissolution and formation of Cu^I^ species from Cu^0^ is regarded as a conclusive hypothesis for the generation of reactive oxygen species responsible for the degradation of organic contaminants under acidic conditions. The contaminant removal efficiencies of nZVC were observed to be increased with increase in dose of nZVC, concentration of oxidizing or reducing agent and application of external energy source, with a significant decrease in reaction time. The novelty of the review article in this study includes the summarization of research results of the various nZVC mediated contaminant degradation studies in tabulated form to provide an opportunity for the researchers to understand the optimum experimental parameter and conditions involved in the pollutant removal process.

## 6 Conclusion and future perspectives

*Nanoscale zerovalent* copper (nZVC) has proved expedient in solving many of the problems related to the existence of organic/inorganic contaminants in the environment and their adverse effects on the ecosystem. Owing to their small size, high surface area, stability associated with their core-shell structure, tendency to degrade organic contaminants under oxic/anoxic conditions either by generation of various reactive oxygen species like H_2_O_2_, O_2_^**.-**^, ^**.**^OH etc. under given reaction conditions, or by reductive removal of contaminants via single or double electron transfer processes made them a suitable candidate for the various studies involving environment remediation processes. However, their stability and activity to control various oxidative/reductive processes largely depends on their storage conditions and surface passivation. The contaminant removal process by nZVC is a pH controlled process, mostly observed in the pH range 3–6, which may be attributed to dissolution of surface oxide layer, conversion of Cu^0^ to Cu^I^ and generation of reactive oxygen species via Fenton-like process under acidic conditions. Alkaline pH results in formation of surface mixed oxide layer and facilitates the contaminant removal process by surface adsorption. The research focused on evaluating the various optimum experimental parameters and conditions to achieve the maximum contaminant removal efficiency from nZVC mediated degradation process. The research also evaluated the role of copper species involved in the catalytic generation of radical species responsible for degradation, and effect of presence of oxidizing and reducing agents.

Further, various materials of synthetic or biogenic origin used as immobilizers or supports may not only provide the stability to the synthesized nano zerovalent copper particles but also significantly alter their physical and chemical properties. Although various supports has been used for the synthesis of nZVC@support based nanocomposite materials, however, the effect of immobilizer on the adsorption and redox properties of nZVC or the origin of synergistic effect when the two worked together for the removal of contaminants has been less studied. In this context, there are significant possibilities to perform research in this field to determine the various activating and stabilizing factors that an immobilizer provides to the synthesized nZVC particles and the underlying mechanism for the contaminant removal via synergistic effect.

## Declarations

### Author contribution statement

All authors listed have significantly contributed to the development and the writing of this article.

### Funding statement

This research did not receive any specific grant from funding agencies in the public, commercial, or not-for-profit sectors.

### Data availability statement

Data included in article/supplementary material/referenced in article.

### Declaration of interests statement

The authors declare no conflict of interest.

### Additional information

No additional information is available for this paper.
